# Leukotriene-modifying agent chemoprophylaxis for severe influenza illness: a multi-approach study

**DOI:** 10.1093/aje/kwag072

**Published:** 2026-04-01

**Authors:** Brittney M Snyder, Corinne A Riddell, Tebeb Gebretsadik, Tan Ding, Rees L Lee, William D Dupont, Justin R Ortiz, Veronika Pav, Thomas J Braciale, Pingsheng Wu, Tina V Hartert

**Affiliations:** Department of Obstetrics and Gynecology, Vanderbilt University Medical Center, Nashville, TN, United States; Divisions of Biostatistics and Epidemiology, School of Public Health, University of California, Berkeley, CA, United States; Department of Biostatistics, Vanderbilt University Medical Center, Nashville, TN, United States; Department of Biostatistics, Vanderbilt University Medical Center, Nashville, TN, United States; Department of Pediatrics, University of Arizona, College of Medicine, Tucson, AZ, United States; Department of Biostatistics, Vanderbilt University Medical Center, Nashville, TN, United States; Center for Vaccine Development & Global Health, University of Maryland School of Medicine, Baltimore, MD, United States; Kennell and Associates Inc., Falls Church, VA, United States; Department of Health Policy and Management, Johns Hopkins Bloomberg School of Public Health, Baltimore, MD, United States; Department of Pathology and Molecular Medicine, University of Virginia, Charlottesville, VA, United States; Department of Biostatistics, Vanderbilt University Medical Center, Nashville, TN, United States; Department of Medicine, Vanderbilt University Medical Center, Nashville, TN, United States; Department of Medicine, Vanderbilt University Medical Center, Nashville, TN, United States; Department of Pediatrics, Vanderbilt University Medical Center, Nashville, TN, United States

**Keywords:** leukotriene-modifying agents, severe influenza illness, chemoprophylaxis, pandemic preparedness, marginal structural model, proportional hazards, case–time–control

## Abstract

Animal and *in vitro* studies suggest that leukotriene-modifying agents (LMAs) may decrease susceptibility to influenza illness. We estimated the effect of LMAs on severe human influenza illness in a retrospective cohort using three analytic approaches: (1) a marginal structural model, (2) a proportional hazards model, and (3) a case–time–control design. Study populations included individuals with asthma and/or allergic rhinitis for whom LMAs are approved and who were enrolled in Tennessee Medicaid (TennCare) or Department of Defense Military Health System (DoD MHS) from 1995 to 2019. Exposed periods were defined by LMA prescription start dates plus days' supply. Severe influenza illness was defined using previously validated International Classification of Diseases criteria. Montelukast accounted for 99% of LMA prescription fills. Adjusted incidence rate ratios from the marginal structural model and adjusted hazard ratios from the proportional hazards model were 1.26 (95% confidence interval [CI], 0.99-1.59) and 1.14 (95% CI, 0.92-1.41) for TennCare and 1.01 (95% CI, 0.84-1.21) and 0.97 (95% CI, 0.82-1.16) for DoD MHS, respectively. Adjusted odds ratios from the case–time–control were 1.11 (95% CI, 0.74-1.65) for TennCare and 1.91 (95% CI, 1.29-2.84) for DoD MHS. Findings in different populations and designs do not support the use of LMAs for chemoprophylaxis of severe influenza illness. Conclusions regarding LMAs other than montelukast were not possible.

## Introduction

Seasonal influenza viruses cause annual epidemics of acute respiratory illness and are associated with substantial morbidity and mortality.^1^^,^^2^ In the United States, influenza is associated with up to 710 000 hospitalizations and 51 000 deaths annually.^3^ Risk groups for severe influenza illness include children <5 years, individuals with asthma or other select chronic diseases, pregnant women, and older adults.^4^

While vaccination remains the best way to prevent severe influenza illness,^5^ new prevention strategies are needed. From the 2004-2005 through 2024-2025 influenza seasons, estimates of influenza vaccine effectiveness to prevent medically attended influenza in the United States ranged from 10% to 60%.^6^ Because of limitations in influenza vaccine performance, the US government has made substantial investments in the development of improved, next-generation influenza vaccines.^7^ A recent expert consultation concluded that the time horizon remains beyond 10 years for new universal vaccine products that may increase vaccine effectiveness and obviate the need for annual vaccination.^8^ Moreover, alternative chemoprophylactic strategies are needed should pandemic strains emerge. Therefore, improving existing vaccine products and developing new preventive measures are urgently needed.^9-11^

Leukotriene modifying agents (LMAs), licensed for the treatment of asthma and allergic rhinitis,^12^ have been shown to reduce influenza virus infection and replication in animal and human cell line models.^13^^,^^14^ The LMA zafirlukast markedly reduced the susceptibility of alveolar epithelial cells to influenza A virus infection and prevented lethal influenza in mice.^13^ The LMA montelukast similarly suppressed influenza A virus infection *in vitro.*^14^ These findings provided the rationale for assessing the potential of LMAs as a novel human chemoprophylactic agent, which is readily available, inexpensive, oral, approved for use in pregnant women and infants 6 months and older, and generally well tolerated.^12^^,^^15^^,^^16^

We hypothesized that individuals treated with LMAs will have reduced risk of severe influenza illness. We designed a cohort including Tennessee Medicaid (TennCare) and Department of Defense Military Health System (DoD MHS) populations to estimate the effect of LMA use on severe influenza illness among children and adults with asthma and/or allergic rhinitis. To increase the robustness of our findings and capitalize on the strengths of multiple statistical methodologies, we used three analytic approaches: (1) marginal structural model (MSM), (2) proportional hazards model, and (3) case–time–control. Table 1 outlines the rationale for each approach.

**Table 1:** Rationale for three complementary approaches to estimate the effect of leukotriene modifying agent (LMA) use on severe influenza illness among children and adults with asthma and/or allergic rhinitis in Tennessee Medicaid and Department of Defense Military Health System populations.

	**Study design and analytic approach**		
	**Marginal structural model (MSM)**	**Proportional hazards model with overlap weights**	**Case*–*time*–c*ontrol**
Rationale for using this approach	MSM was chosen to provide an estimate of the cumulative effect of a time-varying exposure (LMA) on severe influenza illness in the presence of time-varying covariates that may be both confounders and intermediates.	A proportional hazards model was chosen to estimate the effect of LMA use on the hazard rate of severe influenza illness over time. The study was designed to only use parts of an individual’s follow-up such that study confounders cannot be mediators.	The case–time–control design was chosen to control for all known and unknown time-invariant confounders within each person and to account for trends in exposure across the comparison periods (eg, seasonality) by including control subjects whose comparison periods are matched in calendar time to those of case subjects.
Target estimand	The target estimand is a marginal incidence rate ratio comparing the incidence rate of severe influenza illness under a hypothetical strategy in which all individuals received LMAs at all time points versus a strategy where no individuals received LMAs at any time.	The target estimand is a conditional hazard ratio comparing the time to severe influenza illness among a population where everyone uses LMA at all time points versus does not use LMAs at all time points (holding all else constant).	The target estimand is a ratio of ORs comparing the odds of transient LMA use during the 14-day case period versus the 14-day control period among cases, standardized by the corresponding exposure-time trend observed in matched controls.
Strength(s)	An important confounder when assessing the association between LMA use and severe influenza illness is asthma control. As asthma control is a time-varying confounder that is also influenced by prior LMA exposure, it can be appropriately adjusted for using an MSM analysis.	The proportional hazards model allows for the analysis of time-to-event (eg, severe influenza illness) with multiple covariates, including both baseline and time-varying factors. Hazard ratios are commonly estimated parameters that readers are familiar with interpreting.	The case–time–control design estimates the acute effects of LMA use on severe influenza illness. It allows for control of all known and unknown time invariant (constant) confounders within each person, reducing bias from unmeasured confounding.
Limitation(s)	If asthma control is not fully measurable, effect estimates from the MSM analysis may be biased, especially by differences across individuals. MSM analyses are complex to implement, and the parameter estimated is the marginal effect across cumulative LMA doses.	If asthma control is not fully measurable, effect estimates from the proportional hazards model may be biased, especially by differences across individuals.	Time interval of the case or control periods, reference periods, and washout times must be specified *a priori*, and it is possible to not capture a true effect or to estimate a biased effect due to misspecification of these periods.
How other approaches overcome the limitation(s)	The case–time–control analysis eliminates confounding by factors that vary across individuals, such as underlying asthma (or disease) severity. By only using parts of an individual’s follow-up, a simpler proportional hazards analysis (vs. a more complex MSM analysis) with overlap weighting can be performed that overcomes the confounder-as-mediator problem that occurs when we use all of an individual’s follow up where individuals commonly go on and off LMAs repeatedly.	The case–time–control analysis eliminates confounding by factors that vary across individuals, such as underlying asthma (or disease) severity.	MSM and proportional hazards models do not require specification of period time interval.

Three distinct approaches (MSM, proportional hazards model, and case–time–control) were chosen to answer the proposed research question. The interest was in comparing how much the effect estimates would vary across the three approaches, since each method addresses the research question differently. Each approach was chosen to overcome limitations of the other approaches. Consistent results across these approaches increase the robustness of the findings, and deviation may help identify unmet assumptions.

## Methods

### Study design and populations

We utilized a retrospective cohort comprised of two study populations, TennCare (enrollees from January 1, 1995 to December 31, 2019) and the DoD MHS (enrollees October 1, 2002 to September 30, 2018). Details of the study populations are provided in the Supplementary Data (p.6). Both populations included individuals aged 2 to 84 years with asthma and/or allergic rhinitis. Individuals were identified as having asthma and/or allergic rhinitis using International Classification of Diseases (ICD) codes, prescription fills for asthma and allergic rhinitis medications (Tables S1, S2, S3, and S4), and procedure codes (Table S5) or National Drug Codes (NDCs) for allergen immunotherapy (allergic rhinitis only; Table S6). Excluded conditions are listed in Table S7. The study protocol was approved by the Institutional Review Boards of Vanderbilt University Medical Center, Tennessee Department of Health, and Naval Medical Center Portsmouth, Virginia.

### Analytic approaches

#### Marginal structural model

**Figure 1:**
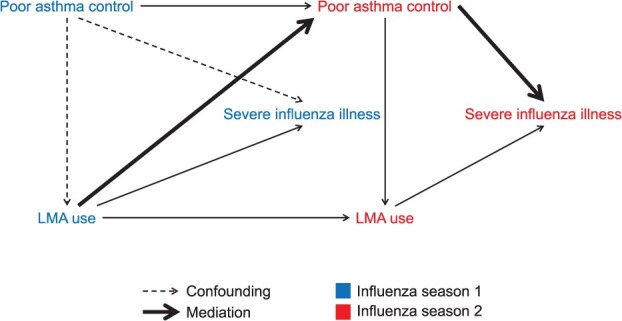
Directed acyclic graph for the MSM analysis, which allows for cumulative effect estimation of a time-varying exposure (LMA use) on severe influenza illness in the presence of time-varying covariates that may be both confounders and intermediate variables (eg, poor asthma control). Abbreviations: LMA, leukotriene-modifying agent; MSM, marginal structural model.

The first approach included an MSM analysis (Figure 1).^17^ We included individuals enrolled during at least one influenza season from 1997-2020 (TennCare) to 2002-2018 (DoD MHS), who had at least 1 year of continuous enrollment prior to the influenza season for accurate covariate capture, and who had LMA exposure in the 365 days prior to at least one influenza season (Figure S1). Of these individuals, some continued LMA use during the influenza season while others discontinued use. These exposed and unexposed periods formed the basis of the comparisons in the analyses.

#### Proportional hazards model

The second approach included a proportional hazards model, which estimated the acute effect of LMAs on severe influenza. The cohort entry eligibility criteria were as above; thus, the sample size and individuals included in the MSM were also included in the proportional hazards model.

#### Case–time–control

The third approach included a case–time–control design.^18^^,^^19^ Within each person, we compared exposure immediately before the outcome (or index) date versus exposure during a prior reference period (Figure S2).^18-20^ For this design, we included individuals with severe influenza illness (cases) identified during 1994-2020 (TennCare) and 2002-2018 (DoD MHS) influenza seasons. Three control persons were randomly selected with replacement for each case from the risk-set of individuals enrolled at the time of the case's hospitalization for severe influenza illness and who did not have severe influenza illness before the case's outcome date. Cases and controls were matched based on asthma and/or allergic rhinitis status at the start of the reference period to ensure similar probabilities of LMA receipt (ie, same drug indication). Both cases and controls must have been continuously enrolled from the start of the reference period to the outcome (or index) date.

### Leukotriene-modifying agent exposure

LMAs were identified from prescription fill NDCs for montelukast, zileuton, or zafirlukast, the three available LMAs in the United States^21^ (Table S4)*.* We defined LMA exposed periods by prescription fill date and days of supply based on no known prolonged effect of LMAs after stopping use and the short half-life.^16^^,^^22^^,^^23^ LMA exposure was defined to start on the fill date and end on the last day of supply (Figure S3). Sequential fills were aggregated into one period if there was ≤7 days between fills. For periods that overlapped, the period start date was defined as the start date of the first LMA prescription fill, and the period end date was defined as the last day of supply for the last prescription fill.

### Severe influenza illness outcome

Our primary outcome was severe influenza illness defined using previously validated criteria as hospitalization with ICD codes for influenza pneumonia, influenza with respiratory insufficiency, or influenza with other non-respiratory illness or organ system involvement.^24^ We required that severe influenza illness events occurred only during influenza seasons, which we defined using data on annual regional influenza circulation (Supplementary Data, Table S8, and Figure S4).

### Statistical analysis

We performed all analyses using R software version 4.4.3 (R Foundation for Statistical Computing, Vienna, Austria) (https://github.com/britt-snyder/LMA-and-Flu). Further details on the study methodology can be found in the Supplementary Data. Results are reported in accordance with RECORD-PE guidelines (Table S9).^25^

#### Marginal structural model

We restricted this analysis to influenza seasons when individuals are at highest risk for severe influenza illness and to avoid misclassification as false-positive influenza tests are more likely during periods of low disease prevalence. Each individual's follow-up time (“person-time”) was divided into analysis periods (Figure S5) to capture changes in LMA use over time, changes in risk factors for severe influenza illness, and fluctuations in enrollment/study inclusion criteria status. Participants were censored when the season ended, enrollment ended, an individual died, or an individual turned age 85 years during the influenza season but before they had a severe influenza illness. Individuals could have multiple periods of varying lengths within the influenza season. Each individual's follow-up time ended once they had a severe influenza illness for that specific influenza season. We used the term “person-seasons” to indicate that an individual may contribute more than one season to the analysis. All analyses were performed separately for each population and meta-analyzed using fixed-effects inverse variance models.

We fit MSMs using a common, two-stage process.^17^^,^^26^ In the first stage, we estimated each individual's period-specific propensity for LMA receipt and not being censored (dependent variables in the models) and used these to calculate stabilized inverse probability of treatment and censoring weights [IPTWs and IPCWs], respectively. Detailed definitions of the model covariates, including prior LMA coverage, sex, race and ethnicity, asthma and/or allergic rhinitis status, acute asthma healthcare utilization, number of asthma encounters, asthma controller medication coverage, allergic rhinitis medication coverage, number of asthma rescue medication fills, age at period state date, age at start of influenza season, Charlson Comorbidity Index, antibiotic use, tobacco use, influenza vaccination, prior influenza vaccination, neuraminidase inhibitor use, nursing home residence, pregnancy, and influenza season, are listed in Tables S10, S11, S12, S13, S14, S15, and S16.

In the second stage, we calculated unadjusted incidence rate ratios (IRRs) and corresponding Wald 95% confidence intervals (CIs) of LMA use on severe influenza illness. We used Poisson regression and robust standard errors, calculated using a sandwich estimator, to account for clustering by individual and included number of days in each period as the offset term for calculating adjusted IRRs (aIRRs) and weights (product of the IPTWs and IPCWs) truncated at the 99th percentile.^27^^,^^28^

We conducted secondary analyses among prespecified groups (<5 years, ≥65 years, those with chronic obstructive pulmonary disease (Table S17), those with allergic rhinitis [without asthma], pregnant individuals, and those who were and were not vaccine-protected) using the same two-stage process outlined in the primary analysis (Supplementary Data and Table S18). Influenza vaccine protection in the current period was defined by CPT and NDC codes (Supplementary Data and Table S15). Vaccine protection was considered to begin 14 days after vaccine receipt.

#### Proportional hazards model

This analysis was also restricted to influenza seasons and follow-up time was defined similarly to the MSM analysis. We additionally divided person-time by underlying risk of influenza based on circulating influenza during the respective season (Figure S6).

Using a similar two-stage process, we first estimated each individual's propensity for LMA receipt using the 20 covariates included in the previous weighting scheme and an additional variable for risk of influenza based on when the period occurred during the respective season. We then used these estimations to calculate overlap weights.^29^ We estimated standardized mean differences (SMD)^30^ to assess imbalances among the unweighted and weighted data, and results were presented as a Love plot.^31^ All variables were balanced (SMD < 0.1^30^) after imposing the overlap weights (Figure S7). We then calculated the unadjusted and adjusted hazard ratios (HR and aHR) and corresponding Wald 95% CIs of LMA use on severe influenza illness using Cox proportional hazards models. Adjusted models included overlap weights and risk of influenza based on when the period occurred during the respective season. The proportional hazards assumption was met in each population.

#### Case–time–control

We ascertained LMA exposure during case or control and reference periods using an approach similar to the methods described by Suissa.^19^ Each case individual contributed one case period and one reference period. Similarly, each control individual contributed one control period and one reference period. The case or control period (14 days) was defined as the “at risk” period directly prior to the severe influenza illness for cases and the corresponding index date for controls. The reference period was a period of the same length (14 days) occurring 30 days prior to the case or control period that provided an estimate of the expected frequency of LMA use for each case and control. LMA exposure was defined as proportion of days on LMAs during the 14-day periods. As most individuals were either never or always on LMAs during the case or control and reference period (Figure S8), we dichotomized this variable using several strategies (Supplementary Data).

We then calculated the matched (on individual) odds ratio (OR) for the cases and controls separately as the ratio of the cases and controls exposed only during the case or control period to the cases and controls exposed only during the reference period (ratio of discordant pairs)^32^ using conditional logistic regression. An OR estimated among the control individuals that was >1 (or <1), signified an increasing (or decreasing) LMA use during follow-up and that this trend introduced a bias in the estimated OR for the case individuals that needed to be removed (Figure S9). To manage this bias, we calculated the case–time–control ratio by including a case status by period interaction term in the model. The estimate for the interaction was equal to the ratio of the OR from the cases to the OR from the controls. By design, all potential confounding characteristics that were stable over the study period were controlled for. However, as asthma control fluctuates over time, we additionally adjusted for this potential time-varying covariate.

To assess potential reverse causation, we performed a *post hoc* sensitivity analysis removing cases with an LMA fill within seven days of hospital admission for severe influenza illness and their matched controls. We performed additional sensitivity analyses varying period widths: (1) shortening case or control and reference periods to 7 days, (2) lengthening case or control and reference periods to 30 days, and (3) lengthening washout time to 60 days (Figure S10).

## Results

### MSM analysis

**Figure 2:**
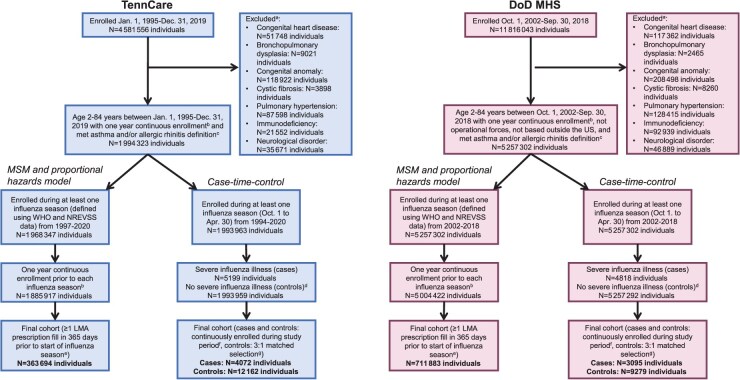
Flow diagram of study populations. (A) Individuals who had one or more of the listed conditions at any point in their life were excluded. Numbers listed are not mutually exclusive. (B) Continuous enrollment defined as ≤31 days of non-enrollment. (C) Asthma definition: ≥1 asthma-related healthcare encounter, ≥2 asthma-specific controller medication prescriptions fills during a two-year time frame, or ≥2 asthma-specific rescue medication prescription fills during a two-year time frame; allergic rhinitis definition: ≥1 allergic rhinitis-related health care encounter, ≥2 montelukast, nasal corticosteroid, nasal mast cell stabilizer, or ophthalmic mast cell stabilizer during a 2-year time frame, ≥2 oral antihistamines/antihistamine-decongestant combinations, nasal antihistamines, or ophthalmic antihistamines during a one-year time frame, ≥1 oral antihistamine/antihistamine-decongestant combination, nasal antihistamine, or ophthalmic antihistamine in addition to at least one dispensing event of montelukast, nasal corticosteroid, nasal mast cell stabilizer, or ophthalmic mast cell stabilizer during a one-year time frame, or ≥1 allergic rhinitis-related procedure or NDCs for allergen immunotherapy. (D) Controls were randomly selected with replacement for each case from the risk-set of individuals who were enrolled at the time of the case’s hospitalization for severe influenza illness and had never had severe influenza illness before the case’s outcome date. (E) Individuals may have multiple periods. (F) Continuous enrollment defined as zero days of nonenrollment. (G) Cases and controls were matched based on asthma and/or allergic rhinitis status at the start of the reference period to ensure similar probabilities of LMA receipt (ie, same drug indication). As individuals could be chosen as a control more than once (chosen with replacement), *n* = 12 162 (TennCare) and *n* = 9279 (DoD MHS) indicate the number of unique individuals. *n* = 4072 (number of cases)*3 = 12 216 controls (TennCare) and *n* = 3095 (number of cases)*3 = 9285 (DoD MHS) were included in the analysis. Abbreviations: DoD MHS, Department of Defense Military Health System; LMA, leukotriene modifying agent; NDC, National Drug Code; NREVSS, National Respiratory Enteric Virus Surveillance System; TennCare, Tennessee Medicaid; MSM, marginal structural model; WHO, World Health Organization.

Of the 4 581 556 individuals enrolled in TennCare, 363 694 (8%) individuals were included in the MSM analysis (Figure 2). This analysis also included 711 883 (6%) DoD MHS enrollees. The median number of influenza seasons during which individuals were enrolled was 2 (interquartile range [IQR], 1-3) for both TennCare and the DoD MHS. The median number of periods per person was 3 (IQR, 1-8) for TennCare and 3 (IQR, 1-7) for the DoD MHS (Table 2).

**Table 2:** Baseline, non-time–varying characteristics of individuals included in the MSM and proportional hazards model analyses.

	**MSM analysis**		**Proportional hazards model analysis**	
**Characteristics**	**TennCare**	**DoD MHS**	**TennCare**	**DoD MHS**
Number of individuals, *N*	363 694	711 883	363 694	711 883
Number of person-seasons^a^^,^^b^, *N*	920 125	1 859 286	920 095	1 859 258
Number of seasons per person, median (IQR)	2 (1-3)	2 (1-3)	2 (1-3)	2 (1-3)
Number of periods^c^, *N*	2 340 952	4 201 265	5 849 774	11 490 761
Number of periods per person^c^, median (IQR)	3 (1-8)	3 (1-7)	10 (5-20)	10 (4-20)
Number of days with LMA use/Total number of days^b^, *N* (%)	40 758 984/161 614 390 (25)	125 311 767/347 461 703 (36)	40 601 646/160 562 495 (25)	125 076 572/346 665 036 (36)
Birth year^d^^,^^e^, *N* (% individuals)				
1913-1925	1707 (0)	621 (0)	1707 (0)	621 (0)
1926-1938	7154 (2)	7256 (1)	7154 (2)	7256 (1)
1939-1951	14 534 (4)	42 751 (6)	14 534 (4)	42 751 (6)
1952-1964	22 683 (6)	104 139 (15)	22 683 (6)	104 139 (15)
1965-1977	26 840 (7)	119 490 (17)	26 840 (7)	119 490 (17)
1978-1990	39 228 (11)	129 644 (18)	39 228 (11)	129 644 (18)
1991-2003	125 295 (34)	195 616 (27)	125 295 (34)	195 616 (27)
2004-2016	126 253 (35)	112 366 (16)	126 253 (35)	112 366 (16)
Race and ethnicity^d^^,^^f^, *N* (% individuals)				
American Indian	571 (0)		571 (0)	
Asian	863 (0)		863 (0)	
Black	63 700 (18)		63 700 (18)	
Hispanic/Latin	2674 (1)		2674 (1)	
Other/unknown	107 819 (30)		107 819 (30)	
Southeast Asian	1294 (0)		1294 (0)	
White	186 773 (51)		186 773 (51)	
Sex^d^^,^^g^, *N* (% individuals)				
Female	199 395 (55)	395 438 (56)	199 395 (55)	395 438 (56)
Male	164 299 (45)	315 465 (44)	164 299 (45)	315 465 (44)
Influenza season^a^^,^^b^^,^^h^, *N* (% person-seasons)				
1997-1998 - 2001-2002	31 560 (3)		31 555 (3)	
2002-2003 - 2006-2007	217 190 (24)	355 915 (19)	217 181 (24)	355 910 (19)
2007-2008 - 2012-2013	256 431 (28)	771 527 (41)	256 428 (28)	771 523 (41)
2013-2014 - 2017-2018	296 890 (32)	731 844 (39)	296 882 (32)	731 825 (39)
2018-2019 - 2019-2020	118 054 (13)		118 049 (13)	

Abbreviations: DoD MHS, Department of Defense Military Health System; IQR, interquartile range; MSM, marginal structural model; TennCare, Tennessee Medicaid.

^a^Each person is counted once for every season they contribute data.

^b^The number of seasons differed between analyses because different approaches were taken in handling the small number of individuals with severe influenza illness in more than one season (*n* < 10 in TennCare and DoD MHS). For the MSM analysis, all severe influenza illnesses for those with an illness in more than one season were included. The proportional hazards model analysis was limited to the first severe influenza illness for the few individuals with more than one illness during enrollment.

^c^The number of periods differed between the analyses as person-time was additionally divided by underlying risk of influenza based on circulating influenza during the respective season in the proportional hazards model analysis.

^d^Defined at enrollment.

^e^Earliest and latest birth years were 1913 and 2016 for TennCare and 1918 and 2014 for the DoD MHS, respectively.

^f^As race and ethnicity were missing for 63% of individuals in the DoD MHS population, this variable was not included in the analyses for this population. Race and ethnicity categories listed were those defined by TennCare. Data used for race and ethnicity come from a third-party data processor that creates mutually exclusive categories of race and ethnicity. This grouping may not reflect other sources of Medicaid data in Tennessee (like T-MSIS).

^g^Data missing for 0.1% of individuals included in the DoD MHS population.

^h^Earliest and latest influenza seasons were 1997-1998 and 2019-2020 for TennCare and 2003-2004 and 2017-2018 for the DoD MHS, respectively.

A lower proportion of days during the influenza season with LMA use was observed for TennCare enrollees compared to DoD MHS enrollees (25% vs. 36%) (Table 2). Nearly all periods with LMA use were for montelukast (TennCare and DoD MHS, 99%). The median length of LMA use during the influenza season was 31 days (IQR, 24-49 days) and 54 days (IQR, 31-91 days) for TennCare and DoD MHS enrollees, respectively. TennCare enrollees were younger than DoD MHS enrollees. There were more females than males for both TennCare and the DoD MHS (TennCare, 55% vs. 45%; DoD MHS, 56% vs. 44%), and about half of the TennCare population was White (51%).

**Figure 3:**
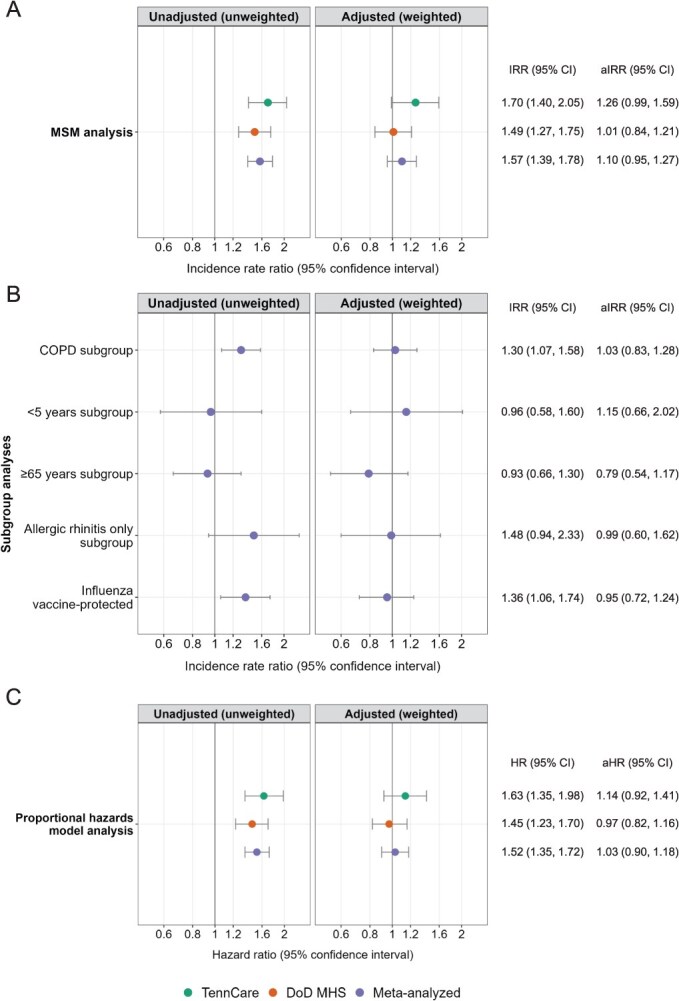
Association between leukotriene modifying agent use and severe influenza illness in (A and B) MSM analysis and (C) proportional hazards model analysis. IRRs were estimated using Poisson regression. aIRRs were estimated using an MSM with inverse probability of treatment and censoring weights. HRs were estimated using Cox proportional hazard models. aHRs included overlap weights. Results were meta-analyzed using fixed-effects inverse variance models. Abbreviations: aHR, adjusted hazard ratio; aIRR, adjusted incidence rate ratio; CI, confidence interval; COPD, chronic obstructive pulmonary disease; DoD MHS, Department of Defense Military Health System; HR, hazard ratio; IRR, incidence rate ratio; TennCare, Tennessee Medicaid.

Among those included in the MSM analysis, 459 severe influenza illnesses occurred among 451 TennCare enrollees (incidence rate/100000 days = 0.28) and 612 severe influenza illnesses occurred among 607 DoD MHS enrollees (incidence rate/100 000 days = 0.18) (Figure S11). After adjusting for confounders and differential loss to follow-up, the aIRR for the estimated effect of LMA on severe influenza illness was 1.26 (95% CI, 0.99-1.59) for TennCare and 1.01 (95% CI, 0.84-1.21) for DoD MHS (Figure 3, panel A and Table S18). The meta-analyzed aIRR was 1.10 (95% CI, 0.95-1.27). For TennCare, we considered that residual confounding may have biased the point estimate away from the null, towards appearing harmful. The estimated E-value^33^^,^^34^ (ie, the strength of the risk ratio needed between an unmeasured confounder with both LMA use and severe influenza illness to shift the point estimate to 1) was 1.83. The effect estimates were similar when restricted to montelukast use only (Table S18).

Among individuals at high risk for severe influenza illness due to age (<5 years and ≥ 65 years), the meta-analyzed aIRRs were 1.15 and 0.79, respectively (<5 years: 95% CI, 0.66-2.02; ≥65 years: 95% CI, 0.54-1.17) (Figure 3, panel B). The meta-analyzed aIRRs for those with COPD and allergic rhinitis only were near 1 (COPD: aIRR, 1.03; 95% CI, 0.83-1.28; allergic rhinitis only: aIRR, 0.99; 95% CI, 0.60-1.62). The meta-analyzed aIRRs for influenza vaccine-protected periods and periods that were not vaccine-protected were 0.95 and 1.12, respectively (vaccine-protected periods: 95% CI, 0.72-1.24; periods that were not vaccine-protected: 95% CI, 0.96-1.30) (Table 3). The effect estimates among DoD MHS enrollees did not differ after excluding active-duty members who are required to receive influenza vaccinations.

**Table 3:** Association between LMA use and severe influenza illness among those who were and were not vaccine-protected during the respective influenza season for the MSM analysis.

**Analysis**	**TennCare**									
	**Sample size (# individuals/# periods)**	**No LMA**			**LMA**			**IRR (95% CI)**	**aIRR (95% CI)**	**Adjusted *P*-value for interaction**
		**Severe influenza illness (periods)**	**Person days**	**Incidence rate/100 000 days**	**Severe influenza illness (Periods)**	**Person-days**	**Incidence rate/100 000 days**			
Vaccine-protected	115 047/389 910	40	19 262 918	0.21	26	7 849 152	0.33	1.34 (0.89-2.00)	1.08 (0.66-1.77)	0.83
Not vaccine-protected	353 066/1 951 042	252	101 592 488	0.25	141	32 909 832	0.43	1.73 (1.41-2.12)	1.27 (1.00-1.62)	
**Analysis**	**DoD MHS**									
	**Sample size (# individuals/# periods)**	**No LMA**			**LMA**			**IRR (95% CI)**	**aIRR (95% CI)**	**Adjusted *P-*value for interaction**
		**Severe influenza illness (periods)**	**Person-days**	**Incidence rate/100 000 days**	**Severe influenza illness (periods)**	**Person-days**	**Incidence rate/100 000 days**			
Vaccine-protected	235 865/652 665	47	35 427 001	0.13	47	22 447 990	0.21	1.37 (1.01-1.87)	0.89 (0.64-1.24)	0.82
Not vaccine-protected	684 715/3 548 600	284	186 426 848	0.15	232	102 729 328	0.23	1.48 (1.25-1.76)	1.03 (0.85-1.25)	
**Analysis**	**DoD MHS (non-active–duty only)**									
	**Sample size (# individuals/# periods)**	**No LMA**			**LMA**			**IRR (95% CI)**	**aIRR (95% CI)**	**Adjusted *P-*value for interaction**
		**Severe influenza illness (periods)**	**Person-days**	**Incidence rate/100 000 days**	**Severe influenza illness (periods)**	**Person-days**	**Incidence rate/100 000 days**			
Vaccine-protected	197 361/567 894	39	30 033 256	0.13	47	19 679 423	0.24	1.44 (1.05-1.96)	0.94 (0.67-1.31)	0.36
Not vaccine-protected	565 153/3 022 180	261	156 942 962	0.17	217	87 096 296	0.25	1.50 (1.25-1.79)	1.03 (0.84-1.26)	
**Analysis**	**TennCare and DoD MHS meta-analyzed**									
	**Sample size (# individuals/# periods**	**No LMA**			**LMA**			**IRR (95% CI)**	**aIRR (95% CI)**	**Adjusted *P-*value for interaction**
		**Severe influenza illness (periods)**	**Person-days**	**Incidence rate/100 000 days**	**Severe influenza illness (periods)**	**Person-days**	**Incidence rate/100 000 days**			
Vaccine-protected	350 912/1 042 575	87	54 689 919	0.16	73	30 297 142	0.24	1.36 (1.06-1.74)	0.95 (0.72-1.24)	N/A
Not vaccine-protected	1 037 781/5 499 642	536	288 019 336	0.19	373	135 639 160	0.27	1.58 (1.38-1.80)	1.12 (0.96-1.30)	

Abbreviations: aIRR, adjusted incidence rate ratio; CI, confidence interval; DoD MHS, Department of Defense Military Health System; IRR, incidence rate ratio; LMA, leukotriene-modifying agent; MSM, marginal structural model; N/A, not applicable; TennCare, Tennessee Medicaid.

Incidence rate ratios for vaccine-protected and not vaccine-protected groups were meta-analyzed using fixed-effects inverse variance models. The adjusted *P-*value for interaction could not be meta-analyzed.

Incidence rate ratios were estimated using Poisson regression. Adjusted incidence rate ratios and corresponding *P-*values were estimated using an MSM with inverse probability of treatment and censoring weights. Weights were calculated adjusting prior LMA coverage, acute asthma healthcare utilization, number of asthma healthcare encounters, asthma controller medication coverage, allergic rhinitis medication coverage, number of asthma rescue medication fills, age at the start of the current period, influenza season, Charlson Comorbidity Index, antibiotic use, tobacco use, influenza vaccination in the 2 months prior to and/or during the influenza season, neuraminidase inhibitor use, nursing home residence, asthma and/or allergic rhinitis status, pregnancy, sex, race, and ethnicity (race and ethnicity were included for TennCare only), and age during the first period of the respective influenza season. Prior LMA coverage, asthma controller medication coverage, allergic rhinitis medication coverage, number of asthma rescue medication fills, age, and Charlson Comorbidity Index were fit with restricted cubic splines. Poisson models included an interaction term (LMA*vaccine-protected) and were adjusted for age during the first period in the respective influenza season, sex, and race and ethnicity (race and ethnicity were included for TennCare only).

Nonactive duty included dependents of active-duty members, dependents of guard/reserve members on active-duty, dependents of retirees, dependent survivors, dependents of inactive guard/reserve members, and retirees.

### Proportional hazards model

Although the same individuals were included in the proportional hazards and MSM analyses, the number of periods was larger for the proportional hazards analysis as we additionally divided person-time by underlying risk of influenza based on circulating influenza within the respective season (Table 2). The aHR for the estimated effect of LMA on severe influenza illness was 1.14 (95% CI, 0.92-1.41) for TennCare and 0.97 (95% CI, 0.82-1.16) for DoD MHS (Figure 3, panel C). The meta-analyzed aHR was 1.03 (95% CI, 0.90-1.18).

### Case–time–control design

The case–time–control design included 4072 case individuals and 12 162 control individuals from TennCare (0.09% and 0.27% of total population, respectively) and 3095 case individuals and 9279 control individuals from the DoD MHS (0.03% and 0.08% of total population, respectively) (Figure 2). Similar to the MSM and proportional hazards analyses, TennCare enrollees were younger than DoD MHS enrollees (Table S19).

**Figure 4:**
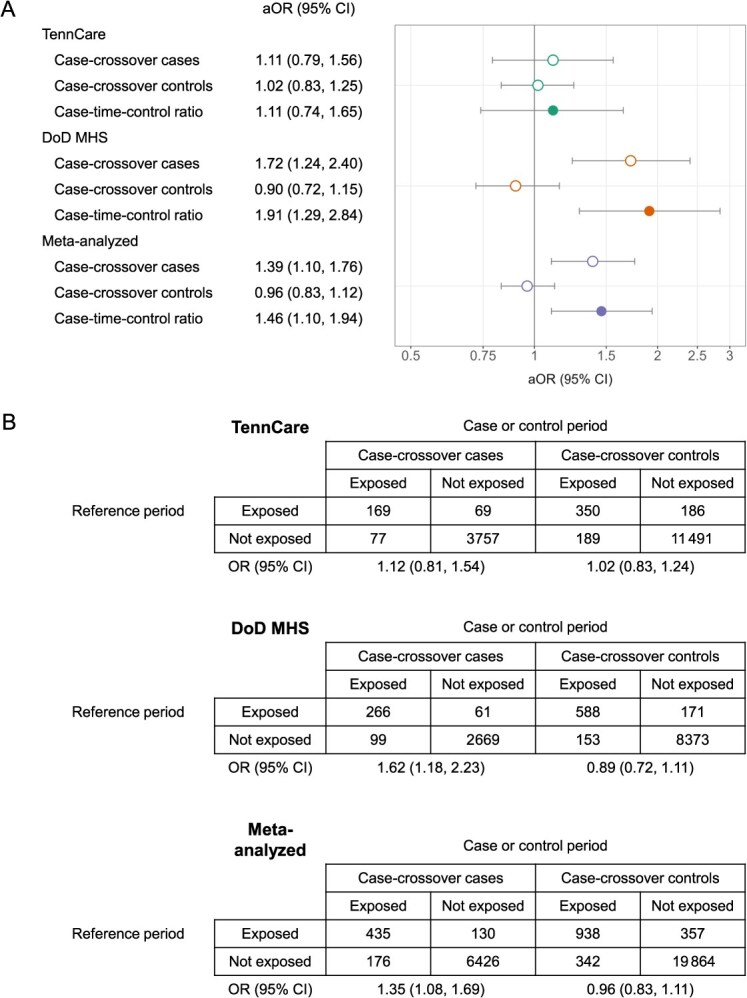
(A) Association between leukotriene modifying agent use and severe influenza illness using the case*–*time*–*control design. (B) 2 × 2 tables of crude OR calculations. Matched (on subject) crude ORs for the case individuals and control individuals were calculated separately as the ratio of the number of case individuals and control individuals exposed only during the case or control period to the number of case individuals and control individuals exposed only during the reference period (ratio of discordant pairs) using conditional logistic regression. The case–time–control ratio was then subsequently calculated by including a case status by period interaction term in the conditional logistic regression model. Adjusted odds ratios were similarly calculated with the addition of variables capturing asthma control. Results were meta-analyzed using fixed-effects inverse variance models. Abbreviations: aOR, adjusted OR; CI, confidence interval; DoD MHS, Department of Defense Military Health System; OR, odds ratio; TennCare, Tennessee Medicaid.

Among case individuals, the frequency of LMA use was higher for case periods than reference periods (TennCare: aOR, 1.11; 95% CI, 0.79-1.56; DoD MHS: OR, 1.72; 95% CI, 1.24-2.40; meta-analyzed: OR, 1.39; 95% CI, 1.10-1.76) (Figure 4). Among control individuals, the frequency was similar (TennCare) or lower (DoD MHS) among control periods than reference periods (TennCare: aOR, 1.02; 95% CI, 0.83-1.25; DoD MHS: aOR, 0.90; 95% CI, 0.72-1.15; meta-analyzed: aOR, 0.96; 95% CI, 0.83-1.12). When we divided the case aOR by the control aOR to account for time trends in LMA use, the frequency of LMA use in case and control periods was elevated compared to the frequency of LMA use in reference periods, though CIs were wide (TennCare: aOR, 1.11; 95% CI, 0.74-1.65; DoD MHS: aOR, 1.91; 95% CI, 1.29-2.84; meta-analyzed: aOR, 1.46; 95% CI, 1.10-1.94). Results were unchanged when applying secondary strategies for dichotomizing LMA exposure (Table S20), removing cases (and their matched controls; 1% of TennCare and DoD MHS) with an LMA fill within seven days of hospital admission for severe influenza illness (Figure S12), and varying period widths (Figure S13).

## Discussion

In this study, we capitalized on the strengths of three complementary analytic approaches to estimate the effect of LMA use on severe influenza illness among individuals within our retrospective cohort with asthma and/or allergic rhinitis enrolled in TennCare and the DoD MHS. Using both the MSM and proportional hazards approaches, the estimated incidence rate ratios and hazard ratios were similar and null for both populations. Under the case–time–control design, the estimated OR was close to null for TennCare and harmful for DoD MHS, with a combined finding also in the direction of LMAs increasing severe influenza illness.

Leukotriene-modifying agents are orally administered pharmacological agents used to block the physiological effects of leukotrienes, inflammatory mediators which cause bronchoconstriction and increased mucus production.^35^ While three generic LMAs are currently available in the US,^21^ montelukast represented 99% of the prescriptions in our study cohorts. The FDA recently released a Boxed Warning for Singulair,^36^ due to neuropsychiatric symptoms appearing among a rare subgroup of users. Thus, risks need to be weighed with potential benefits prior to expanded use of Singular, especially among patients with a family or personal history of depression or suicide.

As cysteinyl leukotrienes contribute to a wide array of respiratory disease pathologies, such as inflammation, thrombosis, fibrotic remodeling, and vascular damage, it has been hypothesized that modification of leukotriene pathways aid in the prevention and treatment of respiratory diseases outside of asthma.^37^ In an animal model of influenza A virus infection, alveolar macrophages prevented lethal infection by suppressing the upregulation of 5-lipoxygenase metabolic pathway genes in alveolar epithelial cells. Treatment with the LMA zafirlukast reduced the susceptibility of alveolar epithelial cells in alveolar macrophage-deficient and wild type infected mice to infection and prevented the development of lethal infection in alveolar macrophage-deficient mice.^13^ Similarly, montelukast has been shown to suppress influenza A virus infection *in vitro*.^14^  *In silico* and *in vitro* studies support the use of montelukast in the treatment of COVID-19.^38^ In an animal model of respiratory syncytial virus infection, montelukast administration prevented airway hyper-responsiveness, inflammation, and vascular permeability.^39^^,^^40^ Montelukast also attenuated lipopolysaccharide-induced lung inflammation in a mouse model of acute respiratory distress syndrome and human neutrophils.^41^ These earlier observations informed our hypothesis that individuals treated with LMAs would have reduced risk of severe influenza illness. Given that our study used observational data, it may be that confounding biased the point estimates. Confounding due to underlying asthma control, or underlying asthma severity, is a concern for all study designs we employed, although this source of confounding is limited in the case–time–control design. LMA exposure may be acting as a surrogate for an asthma exacerbation or allergic rhinitis flare, increasing influenza severity and potentially explaining the harmful direction of the association. The animal study for which our hypothesis was based on^13^ used zafirlukast, not montelukast, and while both are selective cysteinyl leukotriene receptor antagonists, montelukast inhibits at the LTD4 receptor, and zafirlukast inhibits at the LTC4 and LTE4 receptors. Since the vast majority of the prescribed LMAs were montelukast in our study, conclusions about other LMAs like zafirlukast were not possible. Another key difference in our study and prior studies is that we conducted this study among humans and included all viral strains and subtypes to define severe influenza illness.

There is a need to identify novel chemoprophylactic strategies for preventing severe influenza illness, in addition to annual influenza vaccination. Annual vaccination is currently the most effective and evidence-based intervention to prevent seasonal influenza illness and complications.^42^ However, variable vaccine effectiveness and need for annual vaccination necessitate efforts to improve current prevention strategies.^1^ Vaccination in addition to adjunctive chemoprophylaxis may provide improved protection for high-risk populations in whom influenza vaccines are least immunogenic (ie, young children and older adults)^4^ and for all high-risk groups during years when the influenza vaccine has lower effectiveness, or during pandemics. Influenza chemoprophylactic drugs could also be of particular value for pandemic response as they could be stockpiled and deployed rapidly, while specific pandemic influenza vaccines take many months to produce.^1^ While our findings do not support the use of LMAs for prevention of severe influenza illness, our study was limited to individuals with asthma and/or allergic rhinitis prescribed almost exclusively montelukast and, therefore, may not be generalizable to populations without these conditions or to LMAs other than montelukast. Future studies may be warranted to estimate the effect of LMA use on severe and non-severe influenza illness among individuals without asthma and/or allergic rhinitis and using alternative LMAs such as zafirlukast.

We compared findings from three approaches with the intent to overcome the limitations of a single observational study approach. Because TennCare and DoD MHS study populations differed with respect to the age distribution of enrollees and presumed socio-economic differences, it is possible that the true underlying effects of LMA use on severe influenza illness differ between these groups. Results were consistently null across population and methodological approaches. The discrepancy in findings for the case–time–control design within the DoD MHS population may be attributed to misclassification of controls, residual exposure trends, or residual time-varying confounding. We also cannot completely rule out that this could be a spurious finding. Thus, caution should be taken when interpreting these findings.

Our study has many strengths, including application of three rigorous analytic methodologies and large, diverse study populations. To avoid immortal time bias, we used analytic techniques that account for changes in LMA exposure over time. In both healthcare systems, administrative data files and comprehensive outpatient pharmacy claims were linked, allowing us to identify our study populations with asthma and/or allergic rhinitis, LMA prescription fills and date, severe influenza illness, and covariate information. We used a validated algorithm to define severe influenza illness and advanced statistical methods to foster mimicking a randomized clinical trial design and control for time-varying covariates.

Our study also has limitations. LMA prescription fills do not necessarily equate with medication utilization, and we defined LMA exposure periods by fill date and days of supply. Thus, misclassification is possible if individuals did not take their medications once per day after a prescription fill. As severe influenza illness is a rare outcome, we *a priori* designed and powered the study which required the combination of two large populations. The DoD MHS is likely more socio-demographically representative of the US population^43-46^ than TennCare, which primarily insures a vulnerable population of young, low-income individuals in Tennessee. Although the populations are different, we believe they offer comparable internal validity (ie, they offer comparable control for confounding bias, measurement error, and selection bias). The data sources both include comprehensive information on administrative and pharmacy fill data, and variables included in the analyses were similarly ascertained from both data sources. Although we meta-analyzed results to increase the power for all designs/analyses, meta-analysis presumes that the underlying effect is consistent between both populations, which may not be true. Additionally, we did not have the sample size to perform sub-analyses by year or by corresponding major viral strain and subtype. While the ≥65 years subgroup had the most preventive aIRR estimated of all the secondary meta-analyzed MSM analyses, this group had a small sample size as reflected in a wide CI and was one of several subgroup analyses performed. Given the substantial influenza-related morbidity in older adults, further research in this subgroup may be warranted. We focused on the most severe complications of influenza, for which novel chemoprophylactic strategies would have the greatest benefit. Due to potential misclassification (undiagnosed cases) of nonsevere influenza illness, we did not assess this outcome. As administrative datasets may not completely capture influenza vaccines received, vaccine protection may be undermeasured in our secondary MSM analysis. However, immunization billing codes in administrative data have been shown to be concordant with electronic health records where actual vaccine receipt is documented.^47^ We adjusted for asthma control, using variables capturing acute asthma healthcare utilization and number of asthma encounters, as a potential time-varying confounder in all analyses. However, we remain concerned that residual confounding persisted (as suggested by the E-value for TennCare) as asthma control is difficult to define and capture within administrative datasets. The chief concern is differences between individuals in asthma control for MSM and proportional hazards model and varying asthma control over time for each individual for the case–time–control design. One possibility is that residual confounding is responsible for the unexpected harmful effect estimates in all study designs.

### Conclusions

We leveraged complementary and rigorous statistical approaches and two large, diverse study populations to estimate the effect of LMA use on severe influenza illness. Our findings do not support the use of the LMA montelukast for prevention of severe influenza illness among individuals with asthma and/or allergic rhinitis, though conclusions regarding LMAs other than montelukast were not possible. Future studies may be warranted to estimate the effect of LMA use on severe and non-severe influenza illness among individuals without asthma and/or allergic rhinitis and using alternative LMAs such as zafirlukast.

## Supplementary Material

Web_Material_kwag072

## Data Availability

Data from the Division of TennCare in the Tennessee Department of Finance and Administration and the Department of Defense are not publicly available due to privacy reasons. Analytic code is available at: https://github.com/britt-snyder/LMA-and-Flu.
