# Supplementary material for: Leukotriene-modifying agent chemoprophylaxis for severe influenza illness: a multi-approach study
Source: Am J Epidemiol. 2026 Apr 1;195(6):1634–46. doi: 10.1093/aje/kwag072 (PMC13231862; doi:10.1093/aje/kwag072)
Supplement: Web_Material_kwag072 [file web_material_kwag072.zip › Supplementary_Data_with_Author_Byline.docx]

**Leukotriene modifying agent chemoprophylaxis for severe influenza illness:**

**A multi-approach study**

Brittney M. Snyder, Corinne A. Riddell, Tebeb Gebretsadik, Tan Ding, Rees L. Lee, William D. Dupont, Justin R. Ortiz, Veronika Pav, Thomas J. Braciale, Pingsheng Wu, Tina V. Hartert

**Supplementary Data**

[Methods 3](#_Toc208323230)

[Study design and populations 3](#_Toc208323231)

[More information about why we restricted to individuals who had LMA exposure in the 365 days prior to at least one influenza season for the MSM and proportional hazards analyses 5](#_Toc208323232)

[Healthcare systems and data sources 6](#_Toc208323233)

[Leukotriene modifying agent exposure 6](#_Toc208323234)

[Severe influenza illness outcome 7](#_Toc208323235)

[Influenza season identification 8](#_Toc208323236)

[Statistical analysis 10](#_Toc208323237)

[References 21](#_Toc208323238)

[Table S1. 24](#_Toc208323239)

[Tables S2-S4 are provided in the Supplementary Data excel file 28](#_Toc208323240)

[Table S5. 29](#_Toc208323241)

[Table S6 is provided in the Supplementary Data excel file 30](#_Toc208323242)

[Table S7. 31](#_Toc208323243)

[Figure S1. 32](#_Toc208323244)

[Figure S2. 34](#_Toc208323245)

[Figure S3. 35](#_Toc208323246)

[Table S8. 36](#_Toc208323247)

[Figure S4. 37](#_Toc208323248)

[Table S9 39](#_Toc208323249)

[Figure S5 44](#_Toc208323250)

[Table S10. 46](#_Toc208323251)

[Table S11 is provided in the Supplementary Data excel file 52](#_Toc208323252)

[Table S12. 53](#_Toc208323253)

[Tables S13-16 are provided in the Supplementary Data excel file 55](#_Toc208323254)

[Table S17. 56](#_Toc208323255)

[Table S18. 57](#_Toc208323256)

[Figure S6. 60](#_Toc208323257)

[Figure S7. 61](#_Toc208323258)

[Figure S8 62](#_Toc208323259)

[Figure S9. 63](#_Toc208323260)

[Figure S10 64](#_Toc208323261)

[Figure S11 65](#_Toc208323262)

[Figure S12 67](#_Toc208323263)

[Figure S13. 69](#_Toc208323264)

[Table S19 73](#_Toc208323267)

[Table S20 74](#_Toc208323268)

# Methods

## Study design and populations

We utilized a retrospective cohort comprised of two study populations, TennCare (enrollees from January 1, 1995-December 31, 2019) and the DoD MHS (enrollees October 1, 2002-September 30, 2018). Both populations included individuals aged 2-84 years with asthma and/or allergic rhinitis. To ensure adequate data capture, enrollees must have had at least one year of continuous enrollment, were not based outside the US (DoD MHS only), and were not active-duty members assigned to the military’s operational forces (DoD MHS only).

Individuals in our retrospective cohort were identified as having asthma if they had any of the following from age 2-84 years: 1) an inpatient or outpatient visit with an International Classification of Diseases 9^th^ revision (ICD-9) code 493.xx (asthma) or ICD 10^th^ revision (ICD-10) code J45.xx (asthma), 2) at least two separate dispensing events during a two-year time frame of an asthma controller medication (**Table S2**), 3) at least two separate dispensing events during a two-year time frame of an asthma rescue medication (i.e., short-acting beta-agonist [SABA]; **Table S2**). Individuals were defined as having allergic rhinitis if they had any of the following from age 2-84 years: 1) an inpatient or outpatient visit with ICD-9 code 477.xx (allergic rhinitis) or ICD-10 code J30.xx (vasomotor and allergic rhinitis), 2) at least two separate dispensing events during a two-year time frame of montelukast, nasal corticosteroids, nasal mast cell stabilizers, or ophthalmic mast cell stabilizers (**Tables S3 and S4**), 3) at least two separate dispensing events during a one-year time frame of oral antihistamines/antihistamine-decongestant combinations, nasal antihistamines, or ophthalmic antihistamines (**Table S3**), 4) at least one dispensing event of oral antihistamine/antihistamine-decongestant combination, nasal antihistamine, or ophthalmic antihistamine in addition to at least one dispensing event of montelukast, nasal corticosteroid, nasal mast cell stabilizer, or ophthalmic mast cell stabilizer during a one-year time frame (**Tables S3 and S4**), 5) a procedure code (**Table S5**) or national drug code (NDC) for allergen immunotherapy (**Table S6**). Some patients were defined as having asthma or allergic rhinitis based on montelukast use only, since montelukast is used as treatment exclusively for these conditions. For these patients, to differentiate asthmatics and allergic rhinitis patients, we further defined an individual as having allergic rhinitis (but not asthma) if montelukast was filled on or after January 1, 2003, which was when montelukast was approved by the FDA for the treatment of allergic rhinitis (1), and they did not meet criteria for asthma.

Separate dispensing events for all medications, excluding leukotriene modifying agents (LMAs), were defined by prescription fills occurring on separate days. If two prescription fills for NDCs, excluding LMAs, from the same medication category (e.g., inhaled corticosteroids) occurred on the same day, we treated these fills as one event. For prescription fills for LMA NDCs, dispensing events were defined by prescription fills regardless of when they were filled. If two prescription fills for LMA NDCs occurred on the same day, we treated these fills as separate events.

Asthma and allergic rhinitis medication NDCs were first identified through therapeutic class categories in the Tennessee Medicaid (TennCare) dataset. We then pulled all NDCs in corresponding therapeutic classes and with the same product names from the Department of Defense Military Health System (DoD MHS) Data Repository. We curated this list to remove medications that were not indicated for asthma or allergic rhinitis based on physician review. The full lists of NDCs included in the asthma and allergic rhinitis definitions can be found in **Tables S2-S4**.

To avoid misclassification with asthma (as many of these conditions may present with symptoms similar to asthma), individuals were excluded if they ever had an ICD diagnosis of congenital heart disease, bronchopulmonary dysplasia, congenital anomaly, cystic fibrosis, pulmonary hypertension, immunodeficiency, or neurological disorders (**Table S7**).

## More information about why we restricted to individuals who had LMA exposure in the 365 days prior to at least one influenza season for the MSM and proportional hazards analyses

Because marginal structural models (MSM) use inverse probability of treatment weights (IPTWs) that are the inverse of the propensity score (more information provided in the ‘Statistical analysis’ section below), we first examined the probability of LMA receipt based on measured covariates among those who received treatment during the influenza season (regardless of prior LMA receipt) compared to those who never received treatment during the influenza season (regardless of prior LMA receipt). Only 2% (TennCare) and 1% (DoD MHS) of those who never received treatment during the influenza season had more than a 10% probability of receiving an LMA vs. 24% (TennCare) and 18% (DoD MHS) of those who received treatment during the influenza season. Thus, most individuals who never received treatment during the influenza season (regardless of prior LMA receipt) were unlikely to ever receive LMAs based on their measured covariates, making them a distinct population from individuals who received treatment during the influenza season (regardless of prior LMA receipt) and putting analyses of the total cohorts at risk of bias from unmeasured confounding. When the analysis was restricted to individuals with LMA exposure in the 365 days prior to the influenza season, all individuals – including those who did not use LMAs during the influenza season and those who used LMAs during the influenza season – had probabilities of >10% for LMA receipt. Restricting to this population minimized the chance of bias from unmeasured confounding as it made the LMA users and non-users more similar across measured confounders (**Figure S1**).

## Healthcare systems and data sources

TennCare provides care to low-income pregnant women, children, older adults, and individuals with disabilities. It insures approximately 20% of Tennessee’s population, including 50% of its children (2). This data source includes all healthcare encounters and outpatient prescription fills paid through TennCare.

The DoD MHS provides active-duty and retired US military personnel and their dependents expansive medical care and prescription coverage. The DoD MHS Data Repository includes administrative and outpatient pharmacy fill data from healthcare encounters at military hospitals/clinics (i.e., direct care), as well as services provided by civilian facilities where TRICARE, the uniformed services healthcare program, is the sole insurer or cost-shares with other insurers (i.e., private sector care).

## Leukotriene modifying agent exposure

Three generic LMAs are currently available in the US (3). Montelukast and zafirlukast are cysteinyl leukotriene (CysLT_1_) receptor antagonists approved for use in children older than 6 months and five years, respectively (3-5). Montelukast is dosed once daily, requires no monitoring, and can be used during pregnancy (3, 4, 6, 7). Montelukast is also Food and Drug Administration (FDA)-approved for treatment of allergic rhinitis (6). Zileuton acts further upstream in the 5-lipoxygenase (5-LO) pathway to inhibit the formation of CysLTs and other 5-LO metabolites, such as leukotriene B_4_, and is approved for use in children 12 years and older (3, 8). LMAs are generally well-tolerated and safe (3, 9).

LMAs were identified from prescription fill NDCs for montelukast, zileuton, or zafirlukast (**Table S4**)*.* We defined LMA exposed periods by prescription fill date and days of supply based on no known prolonged effect of LMAs after stopping use and the short half-life (4, 5, 8). LMA exposure was defined to start on the fill date and end on the last day of supply (**Figure S3**). Sequential fills were aggregated into one period if there was ≤7 days between fills. For periods that overlapped, the period start date was defined as the start date of the first LMA prescription fill and the period end date was defined as the last day of supply for the last prescription fill.

## Severe influenza illness outcome

Our primary outcome was severe influenza illness defined using previously validated criteria as hospitalization with ICD codes for influenza pneumonia, influenza with respiratory insufficiency, or influenza with other non-respiratory illness or organ system involvement (10). We only included severe influenza illness occurring during influenza seasons (more information provided in the ‘Influenza season identification’ section below) to improve specificity of the outcome. Severe influenza illness was defined as meeting any of the following ICD criteria:

- Influenza pneumonia: One or more ICD-9/10 codes for influenza pneumonia
- Influenza with respiratory insufficiency: One or more ICD-9/10 codes for influenza AND one or more ICD-9/10 codes for acute respiratory distress/failure, respiratory and circulatory disorders, or continuous mechanical ventilation
- Influenza with other non-respiratory illness or organ system involvement: One or more ICD-9/10 codes for influenza AND one or more ICD-9/10 codes for central nervous system disorders, diseases of the digestive or genitourinary system, shock, sepsis, or in-hospital death

## Influenza season identification

Marginal structural model and proportional hazards model. We used influenza positive test data collected by the World Health Organization (WHO) and the National Respiratory Enteric Virus Surveillance System (NREVSS) for public health and clinical laboratories in the United States to define start and end dates for each influenza season. The influenza data in the public domain, and can be obtained from the Centers for Disease Control (<https://www.cdc.gov/fluview/?CDC_AAref_Val=https://www.cdc.gov/flu/weekly/overview>). Although our TennCare population included data from 1995-1997, data were not available for the 1994-1995 to 1996-1997 influenza seasons. Therefore, we were not able to accurately determine start and end dates for these seasons, and we removed them from our analyses. Combined data from public health and clinical laboratories were provided prior to the 2015-2016 influenza season, and we used these combined data for these seasons. As separate data were provided for public health and clinical laboratories for the 2015-2016 to 2019-2020 influenza seasons, we only used data from clinical laboratories to define influenza season start and end dates for these seasons. We aggregated data by Census Region (11) and Morbidity and Mortality Weekly Report (MMWR) week (12) to calculate the influenza test positivity proportion for each Census Region-week. Weeks with fewer tests (in the earliest years of data collection during off-season weeks) generated imprecise estimates of the test positivity rate. Thus, we smoothed these data using a Poisson model of the number of positive tests as a function of a restricted cubic spline for week offset by the log number of tests. We used this model to generate smoothed predictions. Influenza season start dates were defined as the second of two consecutive weeks where model-smoothed test positivity rate was >5%, and influenza season end dates were defined as the second of two consecutive weeks where model-smoothed test positivity rate was <5% (**Figure S4**) (13). Occasionally, the test positivity rate hovered near 5% leading to the detection of “seasons” that were either very close to a longer season or very short in duration. In these cases, we viewed the identified seasonal periods and collapsed with nearby neighboring seasons (with only a very short season in between) or removed very short seasons (where the test positivity only stayed above 5% for a very short period of time). We used influenza season start and end dates from the South Census Region for the TennCare population (**Table S8**). As we were unable to determine where DoD MHS enrollees were located at the start of the influenza season, we used US-wide influenza season start and end dates for this population. US-wide influenza season start and end dates were calculated as the median influenza season start and end dates from all four Census Regions. The median difference in influenza season start date across the Census Regions was 3.5 days (interquartile range [IQR] -10.0, 14.0), and the median difference in influenza season end date was 0 days (IQR -14.9, 10.5).

Case-time-control. To increase our case number and corresponding power, we defined influenza seasons from October 1^st^ through April 30^th^ of each study year (TennCare: 1994-2020; DoD MHS: 2002-2018). This definition is commonly used by the Centers for Disease Control (14), and we previously employed this time frame to define influenza seasons in our validation of severe influenza hospitalizations using ICD criteria (10). Our case number was also larger for this design versus the MSM and proportional hazards analyses because we did not limit to individuals with LMA use in the 365 days prior to at least one influenza season.

## Statistical analysis

Data were prepared using SAS version 9.4 (SAS Institute, Cary, NC, USA). We performed all analyses using R software version 4.4.3 (R Foundation for Statistical Computing, Vienna, Austria) (https://github.com/[private until publication]).

Marginal structural model. The first approach included a MSM analysis, which allowed for cumulative effect estimation of a time-varying exposure (LMA) on severe influenza illness in the presence of time-varying covariates that may be both confounders and intermediate variables (**Figure 1**) (15). We restricted this analysis to influenza seasons when individuals are at highest risk for severe influenza illness and to avoid misclassification as false positive influenza tests are most likely during periods of low disease prevalence. Each individual’s follow-up time (“person-time”) was divided into analysis periods (**Figure S5**) to capture changes in LMA use over time, changes in risk factors for severe influenza illness, and fluctuations in enrollment/study inclusion criteria status. A new period started when any of the following occurred: 1) start of a new influenza season, 2) LMA prescription fill, 3) start of enrollment period, 4) influenza vaccination (i.e., change in severe influenza illness risk), 5) start of pregnancy (i.e., change in severe influenza illness risk), 6) age ≥2 years. A period ended when any of the following occurred: 1) influenza season ended, 2) LMA exposure period ended, 3) enrollment period ended, 4) pregnancy ended (delivery, miscarriage, or abortion), 5) severe influenza illness hospitalization admission, 6) death, 7) 85th birthday. Participants were censored when the season ended, enrollment ended, an individual died, or an individual turned age 85 years during the influenza season but before they had a severe influenza illness. Individuals could have multiple periods of varying lengths within the influenza season. Each individual’s follow-up time ended once they had a severe influenza illness for that specific season as they were considered no longer at risk for the outcome. We assumed that an individual’s risk of severe influenza illness was not affected by whether the individual had had such an event in the previous season. We used the term “person-seasons” to indicate that an individual may contribute more than one season to the analysis. As the number of individuals with severe influenza illness in more than one season was small (n<10 in TennCare and DoD MHS), including these subsequent illnesses in our analyses was unlikely to affect our results. Therefore, for the MSM analysis, we included all severe influenza illnesses for those with an illness in more than one season.

We fit the MSM using a common, two-stage process (16):

1. Estimate each individual’s period-specific propensity for LMA receipt and not being censored (dependent variables in the models) and use these to calculate stabilized IPTWs and IPCWs, respectively
2. Calculate unadjusted (unweighted) and adjusted (weighted) incidence rate ratios (IRRs) and corresponding Wald 95% confidence intervals (CIs) of LMA use on severe influenza illness using Poisson regression, weighted by the product of the IPTWs and IPCWs truncated at the 99^th^ percentile (17, 18)

In the first stage, we calculated stabilized IPTWs and IPCWs for each person-period using standard methods, as described by Fewell and colleagues (15, 16). IPTWs and IPCWs estimate the probability of receiving treatment and not being censored, respectively, based on covariates (both time variant and invariant) collected prior to or at the start of the period. Treatment history (i.e., prior LMA coverage defined as proportion of days covered by LMAs in the 365 days prior to the period start date) was included in the denominator models. As the time-varying aspect of the covariates was between periods (not within periods), each weight was fixed during the period. IPTWs and IPCWs tend to be highly variable with positively skewed distributions. Stabilization can reduce variability, resulting in narrower confidence intervals with better coverage rates (16, 17). To stabilize the weights, we first calculated numerator and denominator models for both the IPTWs and IPCWs using logistic regression. We then divided the respective numerator model by the respective denominator model for each person-period and calculated the cumulative product of the weights for each person within each influenza season. These weights were then multiplied (IPTW*IPCW) for inclusion as weights in the second stage model.

IPTW calculation: In the IPTW numerator models, we regressed LMA use in the current period against the following covariates: non-time varying covariates collected at enrollment (sex and race and ethnicity [race and ethnicity were included in the TennCare analyses, not the DoD MHS because of large missingness (63% of individuals missing race and ethnicity data)]) and age, a baseline time-varying covariate captured during the first period in the respective influenza season (covariate definitions listed in **Table S10**). Age was fit with restricted cubic splines.

IPTW numerator model (R formula specified within logistic regression):

LMA use in the current period ~

sex at enrollment +

race and ethnicity at enrollment (TennCare only) +

spline(age at the start of the respective influenza season)

In the IPTW denominator models, we regressed LMA use in the current period against the following covariates: LMA coverage in the 365 days prior to the current period, non-time varying covariates collected at enrollment (sex and race and ethnicity [race and ethnicity were included for TennCare only]), baseline time-varying covariates captured during the first period in the respective influenza season (age and influenza season), time-varying covariates captured at the start of the current period (age, influenza vaccination, and pregnancy), time-varying covariates captured in the 365 days prior to the current period (acute asthma healthcare utilization [indicator of asthma control], number of asthma healthcare encounters [indicator of asthma control], asthma controller medication coverage, allergic rhinitis medication coverage, number of asthma rescue medication fills, antibiotic use, tobacco use, influenza vaccination, neuraminidase inhibitor use, nursing home residence), and time-varying covariates captured at any time prior to the current period (asthma and/or allergic rhinitis status, Charlson Comorbidity Index) (covariate definitions listed in **Tables S10-S16**). LMA coverage in the 365 days prior to current period, asthma controller medication coverage, allergic rhinitis medication coverage, number of asthma rescue medication fills, age, and Charlson Comorbidity Index were fit with restricted cubic splines.

IPTW denominator model (R formula specified within logistic regression):

LMA use in the current period ~

spline(LMA coverage in the 365 days prior to the current period) +

sex at enrollment +

race and ethnicity at enrollment (TennCare only) +

spline(age at the start of the respective influenza season) +

influenza season during which the period occurs +

spline(age at start of the current period) +

influenza vaccine-protected in the current period +

pregnancy in the current period +

acute asthma healthcare utilization in the 365 days prior to the current period +

number of asthma healthcare encounters in the 365 days prior to the current period +

spline(asthma controller medication coverage in the 365 days prior to the current period) +

spline(allergic rhinitis medication coverage in the 365 days prior to the current period) +

spline(number of asthma rescue medication fills in the 365 days prior to the current period) +

antibiotic use in the 365 days prior to the current period +

tobacco use in the 365 days prior to the current period +

influenza vaccination in the 365 days prior to the current period +

neuraminidase inhibitor use in the 365 days prior to the current period +

nursing home residence in the 365 days prior to the current period +

asthma and/or allergic rhinitis status at any time prior to the current period +

spline(Charlson Comorbidity Index at any time prior to the current period)

The IPCW numerator and denominator models were the same as those specified for IPTWs, except the outcome was no censoring during the current period, LMA use in the current period was included as a covariate in the numerator and denominator models, and influenza season was included as a covariate in the numerator model.

IPCW numerator model (R formula specified within logistic regression):

No censoring during the current period ~

LMA use in the current period +

influenza season during which the period occurs +

all other variables listed in IPTW numerator model

IPCW denominator model (R formula specified within logistic regression):

No censoring during the current period ~

LMA use in the current period +

all other variables listed in IPTW denominator model

In the second stage, we calculated the unadjusted (unweighted) and adjusted (weighted) IRRs and corresponding Wald 95% CIs of LMA use on severe influenza illness using Poisson regression, weighted by the product of the IPTWs and IPCWs truncated at the 99^th^ percentile (17, 18). We used robust standard errors, calculated using a sandwich estimator , to account for clustering by individual; adjusted for age during the first period in the respective influenza season, sex and race and ethnicity (race and ethnicity were included for TennCare only); and included number of days in each period (log-transformed) as the offset term.

R formula specified within Poisson regression:

Severe influenza illness in the current period ~

LMA use in the current period +

spline(age during first period in the respective influenza season) +

sex at enrollment +

race and ethnicity at enrollment (TennCare only) +

offset(log(days in period))

Note: We used the R software package ‘survey’ and functions that accounted for weighting and allowed estimation of the appropriate standard errors (accounting for clustering). We used the ‘svydesign’ function to indicate study IDs (unique ID per person season) and weights (IPTW*IPCW), and the ‘svyglm’ function with family=poisson(link=”log”).

Analyses were performed separately for TennCare and DoD MHS populations. We also meta-analyzed the results using fixed-effects inverse variance models. The combined analysis was meta-analyzed, as opposed to pooling individual-level data from both cohorts, due to the inability to fully harmonize covariates.

We conducted secondary analyses among pre-specified groups using the same two-stage process outlined in the primary analysis. As some groups were small, we presented all secondary analyses as the meta-analyzed results. We assessed the effect of LMA use on severe influenza illness among individuals at increased risk based on age at the start of the period (<5 years and ≥65 years) and those with chronic obstructive pulmonary disease (COPD; definition listed in **Table S17**). These two age groups have the lowest influenza vaccine immunogenicity and may benefit the most from potential adjunctive chemoprophylactic therapy (19). We also assessed the effect of LMA use on severe influenza illness among those with allergic rhinitis (and without asthma) at the start of the period to exclude that the effect of LMAs among those with asthma is exclusively mediated by LMA improvement in lung function or optimization of asthma control. As no pregnant individuals had both LMA use during the influenza season and severe influenza illness within the TennCare population, analysis for this high-risk group was performed in DoD MHS only (**Table S18**).

We also conducted a secondary analysis to assess the effect of LMA use on severe influenza illness among those who were and were not vaccine-protected during the respective influenza season using the same two-stage process outlined in the primary analysis. The IPTW and IPCW numerator and denominator models were the same as those specified in the primary analysis; however, instead of adjusting for influenza vaccination in the current period and in the 365 days prior to the current period in the denominator models, we combined these variables into one “vaccine-protected” variable which equaled 1 when there was influenza vaccination in the two months prior to and/or during the respective influenza season and 0 otherwise. In the second stage, we included a statistical interaction term between the “vaccine-protected” variable and the LMA exposure variable to estimate the effect of LMA use on severe influenza illness among those who were and were not vaccinated using Poisson regression, weighted by the product of the IPTWs and IPCWs, truncated at the 99^th^ percentile, and adjusted for age during the first period in the respective influenza season, sex, and race and ethnicity (race and ethnicity were included for TennCare only).

To assess the robustness of our primary findings to potential unmeasured/uncontrolled confounding, we calculated the E-value (20, 21). The E-value defines the minimum strength of association on the risk ratio scale that an unmeasured confounder(s) would need to have with both LMA use and severe influenza illness to explain away the observed association between LMA use and severe influenza illness. Lastly, we were not able to assess the association between non-montelukast (i.e., zafirlukast or zileuton) use and severe influenza illness as there were <15 severe influenza illnesses in TennCare and DoD MHS combined populations among zafirlukast and zileuton users.

Proportional hazards model. This analysis was also restricted to influenza seasons and follow-up time was defined similarly to the MSM analysis. We additionally divided person-time by underlying risk of influenza based on circulating influenza during the respective season (**Figure S6**). To determine the underlying influenza risk, each week of the influenza season was classified as low, medium, or high risk based on that week’s percentage of positive influenza tests based (see “Influenza season identification” section above). Low, medium, and high risk was defined as <11%, 11-20%, and ≥20% positive. For precision, we restricted this analysis to the first severe influenza illness for the few individuals with more than one illness during enrollment.

Using a similar two-stage process, we first estimated each individual’s propensity for LMA receipt using the 20 covariates included in the previous weighting scheme and an additional variable for risk of influenza based on when the period occurred during the respective season. We then used these estimations to calculate overlap weights (22). We estimated standardized mean differences (SMD) (23) to assess imbalances among the unweighted and weighted data, and results were presented as a Love plot (24). All variables were balanced (SMD <0.1 (23)) after imposing the overlap weights (**Figure S7**). We then calculated the unadjusted and adjusted hazard ratios (HR and aHR) and corresponding Wald 95% CIs of LMA use on severe influenza illness using Cox proportional hazards models. Adjusted models included overlap weights and risk of influenza based on when the period occurred during the respective season. The proportional hazards assumption was met in each population.

Case-time-control design. We also used a case-time-control design, an extension of the case-crossover design that controls for all known and unknown time-invariant confounders within each person and additionally accounts for trends in exposure across the comparison periods (e.g., seasonality) via having control subjects that contribute similar comparison periods (25, 26). This analysis also estimated the short-term effects of LMAs on severe influenza. By way of design, all potential confounding characteristics that were stable over the study period were controlled for. However, as asthma control fluctuates over time, we additionally adjusted for this potential time-varying covariate. Asthma control was defined using two variables: 1) acute asthma healthcare utilization and 2) number of asthma encounters. These variables were similar to those provided in **Table S10** for MSM and proportional hazards analyses. However, they were defined in the 30 days prior to the period start date (as opposed to the 365 days prior to the period start date for the MSM and proportional hazards analyses). For precision, we restricted this analysis to the first severe influenza illness for the few individuals with more than one illness during enrollment.

LMA exposure was defined as proportion of days on LMAs during the 14-day periods (i.e., proportion of days covered [PDC]). This is theoretically a continuous variable. For example, if an individual was exposed to LMAs for 2 out of the 14 days in the case period, their PDC would be 2/14=0.14. In practice, most individuals were either never or always on LMAs during the case or control and reference period (i.e., PDC=0 or PDC=1 for most individuals; **Figure S8**). Therefore, we dichotomized this variable using three strategies: 1) primary strategy: if LMA adherence PDC=0, then 0; all else =1, 2) secondary strategy: if LMA adherence PDC <0.2, then 0; all else=1, 3) secondary strategy: if LMA adherence PDC <0.5, then 0; all else =1.

To assess potential reverse causation, we performed a *post-hoc* sensitivity analysis removing cases with an LMA fill within seven days of hospital admission for severe influenza illness and their matched controls. If these cases had another LMA refill within 30 days of hospital admission, we did not remove them as we considered these prescription refills that happened by chance to be close to the hospital admission. We performed additional sensitivity analyses varying period widths: 1) shortening case or control and reference periods to 7 days, 2) lengthening case or control and reference periods to 30 days, and 3) lengthening washout time to 60 days (**Figure S10**).

# References

1. Montelukast (Singulair) for Perennial Allergic Rhinitis. *Obstet Gynecol* 2006;107(4).

2. Tennessee State Government Division of TennCare. Information and statistics. Available from: <https://www.tn.gov/tenncare/information-statistics.html>. Accessed March 9, 2026.

3. Boyce JA. Antileukotriene agents in the management of asthma. In: UpToDate, Wood RA, Bochner BS (Ed), UpToDate, Waltham, MA. (Accessed on March 19, 2026).

4. Merck Research Laboratories. Singulair (montelukast sodium) tablets. Available from: <https://www.accessdata.fda.gov/scripts/cder/daf/index.cfm?event=overview.process&ApplNo=020829>. Accessed March 9, 2026.

5. AstraZenecca Pharmaceuticals LP. Accolate (zafirlukast) tablet label. Available from: <https://www.accessdata.fda.gov/scripts/cder/daf/index.cfm?event=overview.process&ApplNo=020547>. Accessed March 9, 2026.

6. Scow DT, Luttermoser GK, Dickerson KS. Leukotriene inhibitors in the treatment of allergy and asthma. *Am Fam Physician* 2007;75(1):65–70.

7. Schatz M, Weinberger SE. Management of asthma during pregnany. In: UpToDate, Dixon AE, Lockwood CJ (Ed), UpToDate, Waltham, MA. (Accessed on September 3, 2025).

8. Cornerstone Therapeutics Inc. Zyflo (zileuton) tablet label. Available from: <https://www.accessdata.fda.gov/scripts/cder/daf/index.cfm?event=overview.process&ApplNo=020471>. Accessed March 9, 2026.

9. Choi J, Azmat CE. Leukotriene Receptor Antagonists. [Updated 2023 June 4]. In: StatPearls [Internet]. Treasure Island (FL): StatPearls Publishing; 2022 Jan-. Available from: <https://www.ncbi.nlm.nih.gov/books/NBK554445/>.

10. Snyder BM, Patterson MF, Gebretsadik T, et al. Validation of International Classification of Diseases criteria to identify severe influenza hospitalizations. *Influenza Other Respir Viruses* 2022;16(3):371–5.

11. United States Census Bureau. Geographic levels. Available from: <https://www.census.gov/programs-surveys/economic-census/guidance-geographies/levels.html#par_textimage_34>. Accessed March 9, 2026.

12. Centers for Disease Control and Prevention. Morbidity & Mortality Weekly Reports (MMWR). Available from: https://ndc.services.cdc.gov/wp-content/uploads/MMWR_Week_overview.pdf. Accessed March 9, 2026.

13. Midgley CM, Haynes AK, Baumgardner JL, et al. Determining the Seasonality of Respiratory Syncytial Virus in the United States: The Impact of Increased Molecular Testing. *J Infect Dis* 2017;216(3):345–55.

14. Centers for Disease Control and Prevention . Flu season. <https://www.cdc.gov/flu/about/season.html?CDC_AAref_Val=https://www.cdc.gov/flu/about/season/index.html>. Accessed March 9, 2026.

15. Robins JM, Hernán MÁ, Brumback B. Marginal Structural Models and Causal Inference in Epidemiology. *Epidemiology* 2000;11(5).

16. Fewell Z, Hernán MA, Wolfe F, et al. Controlling for Time-dependent Confounding using Marginal Structural Models. *Stata J* 2004;4(4):402–20.

17. Xiao Y, Moodie EEM, Abrahamowicz M. Comparison of Approaches to Weight Truncation for Marginal Structural Cox Models. *Epidemiol Methods* 2013;2(1):1–20.

18. Cole SR, Hernán MA. Constructing inverse probability weights for marginal structural models. *Am J Epidemiol* 2008;168(6):656–64.

19. Centers for Disease Control and Prevention. Immunogenicity, efficacy, and effectiveness of influenza vaccines. Available from: <https://archive.cdc.gov/#/details?url=https://www.cdc.gov/flu/professionals/acip/immunogenicity.htm>. Accessed March 9, 2026.

20. VanderWeele TJ, Ding P. Sensitivity Analysis in Observational Research: Introducing the E-Value. *Ann Intern Med* 2017;167(4):268–74.

21. Mathur MB, Ding P, Riddell CA, et al. Web Site and R Package for Computing E-values. *Epidemiology* 2018;29(5):e45–e7.

22. Thomas LE, Li F, Pencina MJ. Overlap Weighting: A Propensity Score Method That Mimics Attributes of a Randomized Clinical Trial. *JAMA* 2020;323(23):2417–8.

23. Austin PC. An Introduction to Propensity Score Methods for Reducing the Effects of Confounding in Observational Studies. *Multivariate Behav Res* 2011;46(3):399–424.

24. Ahmed A, Husain A, Love TE, et al. Heart failure, chronic diuretic use, and increase in mortality and hospitalization: an observational study using propensity score methods. *Eur Heart J* 2006;27(12):1431–9.

25. Hallas J, Pottegård A. Use of self-controlled designs in pharmacoepidemiology. *J Intern Med* 2014;275(6):581–9.

26. Suissa S. The case-time-control design. *Epidemiology* 1995;6(3):248–53.

Table S1. Medication classes used to define our study populations with asthma and/or allergic rhinitis.

| **Definition** | **Medication class** | **TennCare therapeutic class** |
| --- | --- | --- |
| Asthma controller medication | Biologic | Omalizumab |
|  |  | Mepolizumab |
|  |  | Dupilumab |
|  |  | Reslizumab |
|  |  | Benralizumab |
|  | Anticholinergic | Ipratropium bromide |
|  |  | Tiotropium bromide |
|  | Anticholinergic-SABA combination | Ipratropium-albuterol |
|  | Inhaled corticosteroid | Flunisolide |
|  |  | Triamcinolone |
|  |  | Budesonide |
|  |  | Mometasone |
|  |  | Fluticasone propionate |
|  |  | Ciclesonide |
|  |  | Beclomethasone |
|  | Inhaled corticosteroid/long-acting beta agonist combination | Mometasone |
|  |  | Fluticasone propionate |
|  |  | Budesonide-formoterol |
|  |  | Ciclesonide |
|  |  | Fluticasone propionate-vilanterol |
|  | Leukotriene modifying agent | Zafirlukast |
|  |  | Zileuton |
|  |  | Montelukast |
|  | Long-acting beta-agonist | Salmeterol xinofoate |
|  |  | Formoterol |
|  |  | Arformoterol |
|  |  | Indacaterol maleate |
|  | Oral mast cell stabilizer | Nedocromil sodium |
|  |  | Cromolyn sodium |
|  | Methylxanthine | Aminophylline |
|  |  | Dyphylline |
|  |  | Theophylline |
| Asthma rescue medication | Short-acting beta-agonist | Bitolterol mesylate |
|  |  | Pirbuterol acetate |
|  |  | Levalbuterol |
|  |  | Metaproterenol sulfate |
|  |  | Albuterol sulfate |
| Allergic rhinitis medication | Leukotriene modifying agent | Montelukast |
|  | Nasal antihistamine | Azelastine hydrochloride |
|  |  | Chlorpheniramine-pseudoephedrine |
|  |  | Phenylephrine |
|  |  | Olopatadine hydrochloride |
|  |  | Azelastine-fluticasone |
|  |  | Diphenhydramine hydrochloride |
|  | Nasal corticosteroid | Budesonide |
|  |  | Mometasone |
|  |  | Fluticasone propionate |
|  |  | Triamcinolone |
|  |  | Ciclesonide |
|  |  | Beclomethasone |
|  |  | Flunisolide |
|  | Nasal mast cell stabilizer | Cromolyn sodium |
|  | Ophthalmic antihistamine | Naphazoline-antazoline |
|  |  | Naphazoline hydrochloride |
|  |  | Naphazoline-pheniramine |
|  |  | Olopatadine |
|  |  | Levocabastine |
|  |  | Emedastine difumarate |
|  |  | Ketotifen |
|  |  | Azelastine hydrochloride |
|  |  | Epinastine hydrochloride |
|  |  | Brompheniramine-phenylephrine |
|  |  | Alcaftadine |
|  |  | Bepotastine besilate |
|  |  | Diphenhydramine hydrochloride |
|  | Ophthalmic mast cell stabilizer | Cromolyn sodium |
|  |  | Nedocromil sodium |
|  |  | Lodoxamide tromethamine |
|  |  | Nedocromil |
|  |  | Pemirolast |
|  | Oral antihistamine/antihistamine-decongestant combination | Clemastine-phenylpropanolamine |
|  |  | Acrivastine |
|  |  | Azatadine-pseudoephedrine |
|  |  | Azelastine hydrochloride |
|  |  | Brompheniramine |
|  |  | Brompheniramine-dextromethorphan |
|  |  | Brompheniramine-phenylephrine |
|  |  | Brompheniramine-phenylpropanolamine |
|  |  | Brompheniramine-phenylpropanolamine-dextromethorphan |
|  |  | Brompheniramine-pseudoephedrine |
|  |  | Carbetapentane |
|  |  | Carbetapentane-brompheniramine |
|  |  | Carbetapentane-chlorpheniramine |
|  |  | Carbetapentane-dexchlorpheniramine |
|  |  | Carbetapentane-diphenhydramine |
|  |  | Carbetapentane-guaifenesin |
|  |  | Carbetapentane-phenylephrine |
|  |  | Carbetapentane-pseudoephedrine |
|  |  | Carbetapentane-pyrilamine |
|  |  | Carbinoxamine |
|  |  | Carbinoxamine-phenylephrine |
|  |  | Carbinoxamine-pseudoephedrine |
|  |  | Cetirizine hydrochloride |
|  |  | Cetirizine hydrochloride-pseudoephedrine |
|  |  | Chlorcyclizine-chlophedianol |
|  |  | Chlorcyclizine-phenylephrine |
|  |  | Chlorcyclizine-pseudoephedrine |
|  |  | Chlorpheniramine maleate |
|  |  | Chlorpheniramine-dextromethorphan |
|  |  | Chlorpheniramine-methscopolamine |
|  |  | Chlorpheniramine-phenylephrine |
|  |  | Chlorpheniramine-phenylpropanolamine |
|  |  | Chlorpheniramine-phenylpropanolamine-phenylephrine |
|  |  | Chlorpheniramine-pseudoephedrine |
|  |  | Clemastine |
|  |  | Cyproheptadine hydrochloride |
|  |  | Desloratadine-pseudoephedrine |
|  |  | Desloratadine |
|  |  | Dexbrompheniramine |
|  |  | Dexbrompheniramine-brompheniramine |
|  |  | Dexbrompheniramine-chlophedianol |
|  |  | Dexbrompheniramine-phenylephrine |
|  |  | Dexbrompheniramine-pseudoephedrine |
|  |  | Dexchlorpheniramine |
|  |  | Dexchlorpheniramine-phenylephrine |
|  |  | Dexchlorpheniramine-pseudoephedrine |
|  |  | Dextromethorphan-chlorpheniramine |
|  |  | Dextromethorphan-diphenhydramine |
|  |  | Dextromethorphan-phenylpropanolamine |
|  |  | Dextromethorphan-phenylpropanolamine-guaifenesin |
|  |  | Dextromethorphan-pseudoephedrine |
|  |  | Diphenhydramine |
|  |  | Diphenhydramine hydrochloride |
|  |  | Diphenhydramine tannate |
|  |  | Diphenhydramine-acetaminophen |
|  |  | Diphenhydramine-phenylephrine |
|  |  | Diphenhydramine-pseudoephedrine |
|  |  | Doxylamine succinate |
|  |  | Doxylamine-dextromethorphan |
|  |  | Doxylamine-phenylephrine |
|  |  | Doxylamine-pseudoephedrine |
|  |  | Fexofenadine |
|  |  | Fexofenadine-pseudoephedrine |
|  |  | Hydroxyzine |
|  |  | Levocetrizine |
|  |  | Loratadine |
|  |  | Loratadine-pseudoephedrine |
|  |  | Phenylephrine |
|  |  | Phenylephrine-potassium |
|  |  | Phenyltoloxamine |
|  |  | Phenyltoloxamine-acetaminophen |
|  |  | Pseudoephedrine-chlophedianol |
|  |  | Pseudoephedrine-methscopolamine |
|  |  | Pyrilamine |
|  |  | Pyrilamine-acetaminophen |
|  |  | Pyrilamine-chlophedianol |
|  |  | Pyrilamine-dexbrompheniramine |
|  |  | Pyrilamine-dextromethorphan |
|  |  | Pyrilamine-pheniramine-phenylephrine |
|  |  | Pyrilamine-phenylephrine |
|  |  | Pyrilamine-phenylephrine-dextromethorphan |
|  |  | Pyrilamine-phenylpropanolamine-pheniramine |
|  |  | Pyrilamine-pseudoephedrine |
|  |  | Terfenadine |
|  |  | Thonzylamine-chlophedianol |
|  |  | Thonzylamine-phenylephrine |
|  |  | Tranilast |
|  |  | Tripelennamine hydrochloride |
|  |  | Triprolidine |
|  |  | Triprolidine-pseudoephedrine |

Tables S2-S4 are provided in the Supplementary Data excel file**.**

Table S5. Procedure codes included in allergic rhinitis definition.

| **Procedure code type** | **Procedure code** | **Definition** |
| --- | --- | --- |
| CPT® | 95004 | Allergen testing procedure |
|  | 95017 | Allergen testing procedure |
|  | 95018 | Allergen testing procedure |
|  | 95024 | Allergen testing procedure |
|  | 95027 | Allergen testing procedure |
|  | 95028 | Allergen testing procedure |
|  | 95044 | Allergen testing procedure |
|  | 95052 | Allergen testing procedure |
|  | 95056 | Allergen testing procedure |
|  | 95070 | Allergen testing procedure |
|  | 95071 | Allergen testing procedure |
|  | 95076 | Ingestion challenge allergy and clinical immunology testing procedure |
|  | 95079 | Ingestion challenge allergy and clinical immunology testing procedure |
|  | 95115 | Allergen immunotherapy service/procedure |
|  | 95117 | Allergen immunotherapy service/procedure |
|  | 95120 | Allergen immunotherapy service/procedure |
|  | 95125 | Allergen immunotherapy service/procedure |
|  | 95130 | Allergen immunotherapy service/procedure |
|  | 95131 | Allergen immunotherapy service/procedure |
|  | 95132 | Allergen immunotherapy service/procedure |
|  | 95133 | Allergen immunotherapy service/procedure |
|  | 95134 | Allergen immunotherapy service/procedure |
|  | 95144 | Allergen immunotherapy service/procedure |
|  | 95145 | Allergen immunotherapy service/procedure |
|  | 95146 | Allergen immunotherapy service/procedure |
|  | 95147 | Allergen immunotherapy service/procedure |
|  | 95148 | Allergen immunotherapy service/procedure |
|  | 95149 | Allergen immunotherapy service/procedure |
|  | 95165 | Allergen immunotherapy service/procedure |
|  | 95170 | Allergen immunotherapy service/procedure |
|  | 95180 | Allergen immunotherapy service/procedure |
|  | 95199 | Allergen immunotherapy service/procedure |
| ICD-9 | 99.12 | Immunization for allergy |
| ICD-10-PCS | 3E013GC | Introduction of other therapeutic substance into subcutaneous tissue, percutaneous approach |
|  | 3E023GC | Introduction of other therapeutic substance into muscle, percutaneous approach |

CPT®, Current Procedural Terminology; ICD-9, International Classification of Diseases, 9^th^ Revision; ICD-10-PCS, International Classification of Diseases, 10^th^ Revision, Procedure Coding System.

Table S6 is provided in the Supplementary Data excel file**.**

Table S7. International Classification of Diseases (ICD) codes used to identify excluded conditions.

| **Exclusion** | **ICD-9 code** | **ICD-10 codes** |
| --- | --- | --- |
| Congenital heart disease | 746.xx (other congenital anomalies of heart) | Q20.x-Q28.x (congenital malformations of the circulatory system) |
| Bronchopulmonary dysplasia | 770.7 (chronic respiratory diseases arising in the perinatal period) | P27.1 (bronchopulmonary dysplasia originating in the perinatal period) |
| Congenital anomaly | 745.xx (bulbus cordis anomalies and anomalies of cardiac septal closure)  746.xx (other congenital anomalies of heart)  747.xx (other congenital anomalies of circulatory system)  748.xx (congenital anomalies of respiratory system)  758.xx (chromosomal anomalies) | Q20.x (congenital malformations of cardiac chambers and connections)  Q21.xx (congenital malformations of cardiac septa)  Q22.x (congenital malformations of pulmonary and tricuspid valves)  Q23.x (congenital malformations of aortic and mitral valves)  Q24.x (other congenital malformations of heart)  Q25.xx (congenital malformations of great arteries)  Q26.x (congenital malformations of great veins)  Q31.x (congenital malformations of larynx)  Q32.x (congenital malformations of trachea and bronchus)  Q33.x (congenital malformations of lung)  Q34.x (other congenital malformations of respiratory system)  Q90.x (Down syndrome) |
| Cystic fibrosis | 277.0 (cystic fibrosis) | E84.xx (cystic fibrosis) |
| Pulmonary hypertension | 415.0 (acute cor pulmonale)  416.x (chronic pulmonary heart disease)  417.8 (other specified diseases of pulmonary circulation)  417.9 (unspecified disease of pulmonary circulation) | I27.xx (other pulmonary heart diseases) |
| Immunodeficiency | 279.xx (disorders involving the immune mechanism) | D80.x-D89.xxx (certain disorders involving the immune mechanism) |
| Neurological disorder | 343.x (infantile cerebral palsy)  359.xx (muscular dystrophies and other myopathies) | G80.x (cerebral palsy)  G71.xxxx (primary disorders of muscles) |

Figure S1. Rationale for restricting to individuals who had LMA exposure in the 365 days prior to the influenza season for the marginal structural model and proportional hazards model. This figure depicts the probability of leukotriene modifying agent (LMA) receipt for the 2009 pandemic influenza season among the A) total TennCare population, B) TennCare population restricted to individuals who had LMA exposure in the 365 days prior to the influenza season, C) total DoD MHS population, and D) DoD MHS population restricted to individuals who had LMA exposure in the 365 days prior to the influenza season.


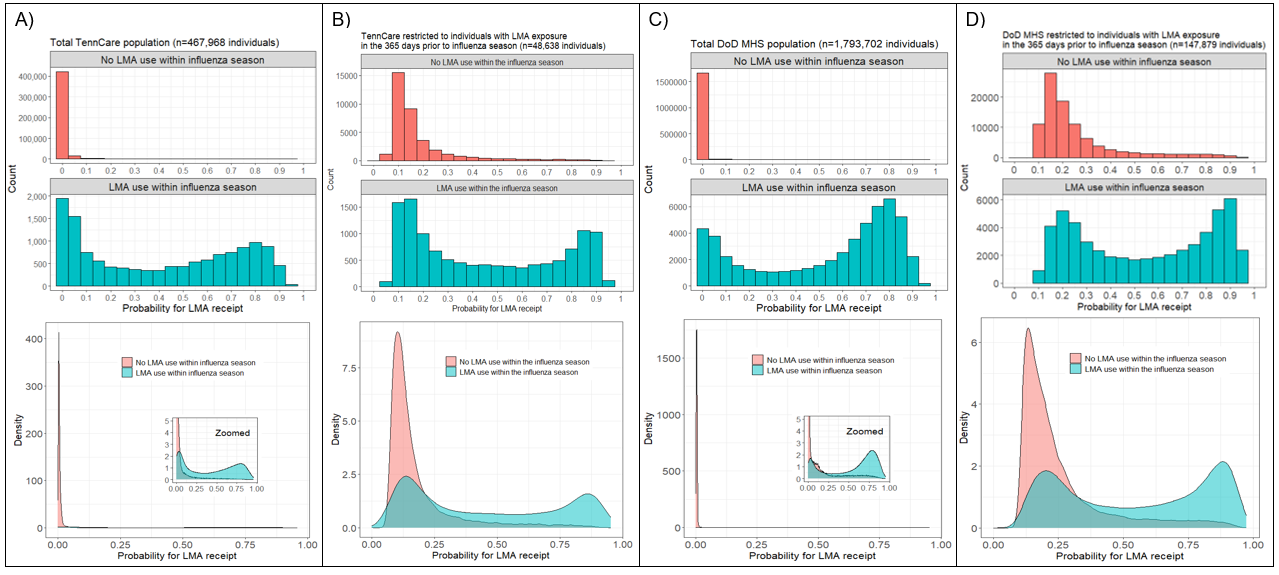


Analyses restricted to the first period per individual. Probabilities were calculated using logistic regression, adjusting for prior LMA coverage, acute asthma healthcare utilization, number of asthma healthcare encounters, asthma controller medication coverage, allergic rhinitis medication coverage, number of asthma rescue medication fills, age at the start of the current period, Charlson Comorbidity Index, antibiotic use, tobacco use, influenza vaccination in the current period, prior influenza vaccination, neuraminidase inhibitor use, nursing home residence, asthma and/or allergic rhinitis status, pregnancy, sex, race and ethnicity (race and ethnicity were included for TennCare only), and age during the first period of the respective influenza season. Prior LMA coverage, asthma controller medication coverage, allergic rhinitis medication coverage, and age were fit with restricted cubic splines in the analyses among the total TennCare population, TennCare population restricted to individuals who had LMA exposure in the 365 days prior to the influenza season, total DoD MHS population, and DoD MHS population restricted to individuals who had LMA exposure in the 365 days prior to the influenza season. Number of asthma rescue medication fills was fit with restricted cubic splines in the analyses among TennCare and DoD MHS populations restricted to individuals who had LMA exposure in the 365 days prior to the influenza season. Number of asthma rescue medication fills was categorized (0, 1, 2, 3, 4, 5, 6, 7, 8, 9, 10+) in the analyses among the total DoD MHS population and total TennCare population. Charlson Comorbidity Index was fit with restricted cubic splines in the total TennCare population, total DoD MHS population, and DoD MHS population restricted to individuals who had LMA exposure in the 365 days prior to the influenza season. Charlson Comorbidity Index was categorized (0, 1, 2, 3+) in the TennCare population restricted to individuals who had LMA exposure in the 365 days prior to the influenza season.

Figure S2. Ascertainment of case or control and reference periods for the case-time-control design.


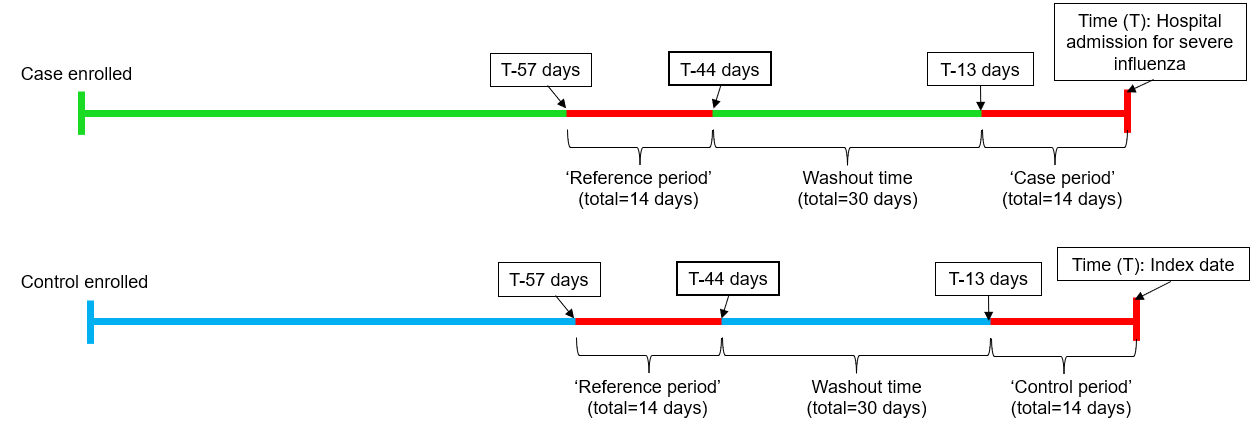


Figure S3. Examples of leukotriene modifying agent (LMA) exposure periods.

**
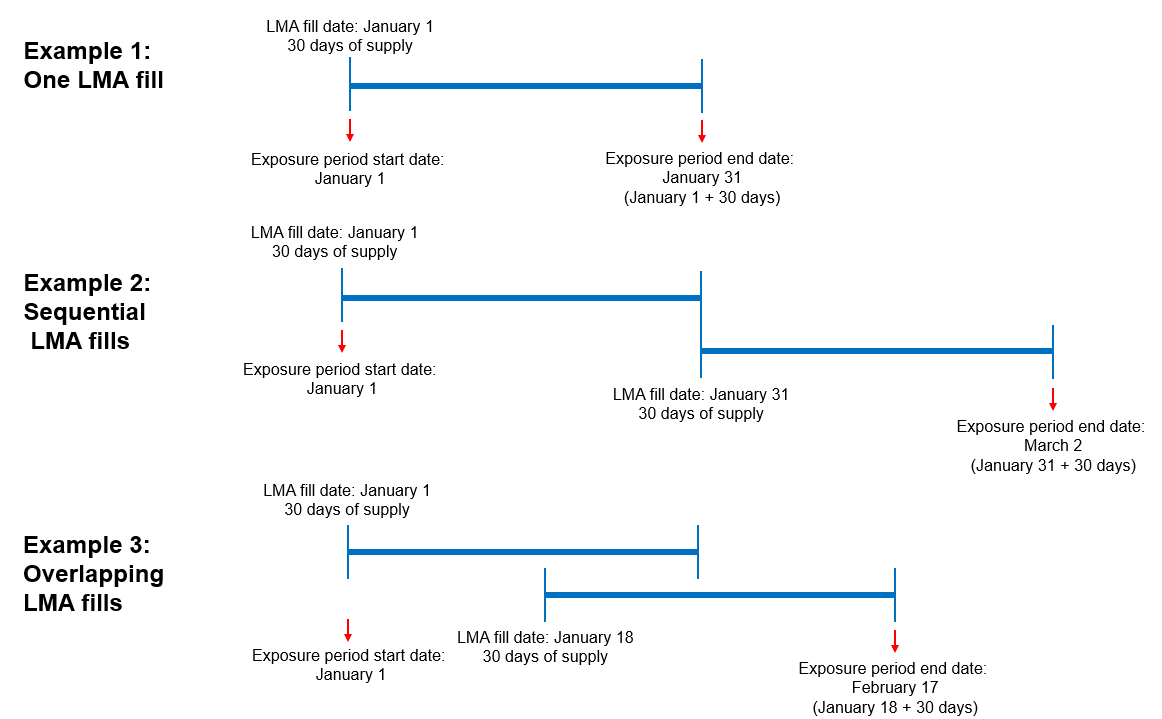
**

Table S8. Influenza season start and end dates for the South Census Region (TennCare) and US-wide (DoD MHS) utilized in the marginal structural model and proportional hazards model.

| **TennCare** | | **DoD MHS** | |
| --- | --- | --- | --- |
| **Influenza season** | **Start and end dates** | **Influenza season** | **Start and end dates** |
| 1997-1998 | Dec. 21-Mar. 29 | -- | -- |
| 1999 | Jan. 10-April 18 | -- | -- |
| 1999-2000 | Dec. 5-Mar. 5 | -- | -- |
| 2000-2001 | Nov. 26-Mar. 18 | -- | -- |
| 2001-2002 | Dec. 30-May 12 | -- | -- |
| 2002-2003 | Dec. 15-Mar. 30 | -- | -- |
| 2003-2004 | Sep. 28-Feb. 1 | 2003-2004 | Oct. 22-Feb. 1 |
| 2005 | Jan. 2-Apr. 24 | 2004-2005 | Dec. 19-May 4 |
| 2005-2006 | Dec. 18-Jun. 11 | 2005-2006 | Dec. 21-May 24 |
| 2006-2007 | Nov. 5-May 13 | 2006-2007 | Dec. 13-May 9 |
| 2007-2008 | Dec. 16-May 4 | 2007-2008 | Dec. 23-May 7 |
| 2008-2010 | Dec. 28-Apr. 11 | 2009-2010 | Jan. 4-Feb 14 |
| 2010-2011 | Oct. 24-Apr. 10 | 2010-2011 | Nov. 14-Apr. 24 |
| 2012 | Feb. 5-Jul. 29 | 2012 | Jan. 22-Jul. 18 |
| 2012-2013 | Oct. 14-May 5 | 2012-2013 | Oct. 28-Jun. 2 |
| 2013-2014 | Aug. 25-Jul. 6 | 2013-2014 | Oct. 16-Jul. 20 |
| 2014-2015 | Oct. 12-Apr. 26 | 2014-2015 | Nov. 2-May 31 |
| 2016 | Jan. 24-Jun. 12 | 2016 | Jan. 24-May 29 |
| 2016-2017 | Dec. 4-Jun. 25 | 2016-2017 | Dec. 18-May 31 |
| 2017-2018 | Nov. 12-May 6 | 2017-2018 | Nov. 26-May 9 |
| 2018-2019 | Nov. 18-May 19 | -- | -- |
| 2019-2020 | Nov. 3-Mar. 29 | -- | -- |

--Earliest and latest influenza seasons were 2003-2004 and 2017-2018 for the DoD MHS.

Figure S4. Defining influenza season start and end dates by Census Region for the marginal structural model and proportional hazards model.

Influenza season start and end dates were defined using influenza positive test data collected by the World Health Organization (WHO) and the National Respiratory Enteric Virus Surveillance System (NREVSS) for public health and clinical laboratories in the United States. Combined data from public health and clinical laboratories were provided prior to the 2015-2016 season, and we used these combined data for these seasons. Data from clinical laboratories were used to define influenza season start and end dates for the 2015-2016 to 2019-2020 seasons as separate data were provided for public health and clinical laboratories for these seasons. Data were aggregated by Census Region and Morbidity and Mortality Weekly Report (MMWR) week to calculate the test positivity proportion for each Census Region-week. Data were smoothed using a Poisson model of the number of positive tests as a function of a restricted cubic spline for week offset by the log number of tests. Influenza season start dates (green vertical lines) were defined as the second of two consecutive weeks where model-smoothed test positivity rate was >5% and influenza season end dates (red vertical lines) were defined as the second of two consecutive weeks where model-smoothed test positivity rate was <5%. Occasionally, the test positivity rate hovered near 5% leading to the detection of “seasons” that were either very close to a longer season or very short in duration. In these cases, identified seasonal periods were viewed and collapsed with nearby neighboring seasons (with only a very short season in between) or very short seasons were removed (where the test positivity only stayed above 5% for a very short period of time). Influenza season start and end dates from the South Census Region were used for the TennCare population, and US-wide influenza season start and end dates were used for the DoD MHS population. US-wide influenza season start and end dates were identified by the median influenza season start and end dates from all four Census Regions.

Table S9**.** The RECORD statement for pharmacoepidemiology (RECORD-PE) checklist of items, extended from the STROBE and RECORD statements, which should be reported in non-interventional pharmacoepidemiological studies using routinely collected health data.

| **Item No** | **STROBE items** | **RECORD items** | **RECORD-PE items** | **Page No** |
| --- | --- | --- | --- | --- |
| **Title and abstract** | | | | |
| 1 | (a) Indicate the study’s design with a commonly used term in the title or the abstract.  (b) Provide in the abstract an informative and balanced summary of what was done and what was found. | 1.1: The type of data used should be specified in the title or abstract. When possible, the name of the databases used should be included.  1.2: If applicable, the geographical region and timeframe within which the study took place should be reported in the title or abstract.  1.3: If linkage between databases was conducted for the study, this should be clearly stated in the title or abstract. | — | 1-2 |
| **Introduction** | | | | |
| Background rationale | | | | |
| 2 | Explain the scientific background and rationale for the investigation being reported. | — | — | 3-4 |
| Objectives | | | | |
| 3 | State specific objectives, including any prespecified hypotheses. | — | — | 4 |
| **Methods** | | | | |
| Study design | | | | |
| 4 | Present key elements of study design early in the paper. | — | 4.a: Include details of the specific study design (and its features) and report the use of multiple designs if used.  4.b: The use of a diagram(s) is recommended to illustrate key aspects of the study design(s), including exposure, washout, lag and observation periods, and covariate definitions as relevant. | 4-10, Figure S2, Figure S5 |
| Setting | | | | |
| 5 | Describe the setting, locations, and relevant dates, including periods of recruitment, exposure, follow-up, and data collection. | — | — | 4-6 |
| Participants | | | | |
| 6 | (a) Cohort study—give the eligibility criteria, and the sources and methods of selection of participants. Describe methods of follow-up. Case-control study—give the eligibility criteria, and the sources and methods of case ascertainment and control selection. Give the rationale for the choice of cases and controls. Cross sectional study—give the eligibility criteria, and the sources and methods of selection of participants.  (b) Cohort study—for matched studies, give matching criteria and number of exposed and unexposed. Case-control study—for matched studies, give matching criteria and the number of controls per case. | 6.1: The methods of study population selection (such as codes or algorithms used to identify participants) should be listed in detail. If this is not possible, an explanation should be provided.  6.2: Any validation studies of the codes or algorithms used to select the population should be referenced. If validation was conducted for this study and not published elsewhere, detailed methods and results should be provided.  6.3: If the study involved linkage of databases, consider use of a flow diagram or other graphical display to demonstrate the data linkage process, including the number of individuals with linked data at each stage. | 6.1.a: Describe the study entry criteria and the order in which these criteria were applied to identify the study population. Specify whether only users with a specific indication were included and whether patients were allowed to enter the study population once or if multiple entries were permitted. See explanatory document for guidance related to matched designs. | 4-10, Supplementary Data |
| Variables | | | | |
| 7 | Clearly define all outcomes, exposures, predictors, potential confounders, and effect modifiers. Give diagnostic criteria, if applicable. | 7.1: A complete list of codes and algorithms used to classify exposures, outcomes, confounders, and effect modifiers should be provided. If these cannot be reported, an explanation should be provided. | 7.1.a: Describe how the drug exposure definition was developed.  7.1.b: Specify the data sources from which drug exposure information for individuals was obtained.  7.1.c: Describe the time window(s) during which an individual is considered exposed to the drug(s). The rationale for selecting a particular time window should be provided. The extent of potential left truncation or left censoring should be specified.  7.1.d: Justify how events are attributed to current, prior, ever, or cumulative drug exposure.  7.1.e: When examining drug dose and risk attribution, describe how current, historical or time on therapy are considered.  7.1.f: Use of any comparator groups should be outlined and justified.  7.1.g: Outline the approach used to handle individuals with more than one relevant drug exposure during the study period. | 8, Supplementary Data, Table S4, Figure S3, Tables S10-S16 |
| Data sources/measurement | | | | |
| 8 | For each variable of interest, give sources of data and details of methods of assessment (measurement). Describe comparability of assessment methods if there is more than one group. | — | 8.a: Describe the healthcare system and mechanisms for generating the drug exposure records. Specify the care setting in which the drug(s) of interest was prescribed. | Supplementary Data |
| Bias | | | | |
| 9 | Describe any efforts to address potential sources of bias. | — | — | 4-13, Supplementary Data |
| Study size | | | | |
| 10 | Explain how the study size was arrived at. | — | — | N/A |
| Quantitative variables | | | | |
| 11 | Explain how quantitative variables were handled in the analyses. If applicable, describe which groupings were chosen, and why. | — | — | Table S10 |
| Statistical methods | | | | |
| 12 | (a) Describe all statistical methods, including those used to control for confounding.  (b) Describe any methods used to examine subgroups and interactions.  (c) Explain how missing data were addressed.  (d) Cohort study—if applicable, explain how loss to follow-up was addressed. Case-control study—if applicable, explain how matching of cases and controls was addressed. Cross sectional study—if applicable, describe analytical methods taking account of sampling strategy.  (e) Describe any sensitivity analyses. | — | 12.1.a: Describe the methods used to evaluate whether the assumptions have been met.  12.1.b: Describe and justify the use of multiple designs, design features, or analytical approaches. | 7-10, Supplementary Data |
| Data access and cleaning methods | | | | |
| 12 | — | 12.1: Authors should describe the extent to which the investigators had access to the database population used to create the study population.  12.2: Authors should provide information on the data cleaning methods used in the study. | — | Supplementary Data |
| Linkage | | | | |
| 12 | — | 12.3: State whether the study included person level, institutional level, or other data linkage across two or more databases. The methods of linkage and methods of linkage quality evaluation should be provided. | — | Supplementary Data |
| **Results** | | | | |
| Participants | | | | |
| 13 | (a) Report the numbers of individuals at each stage of the study (eg, numbers potentially eligible, examined for eligibility, confirmed eligible, included in the study, completing follow-up, and analysed).  (b) Give reasons for non-participation at each stage.  (c) Consider use of a flow diagram. | 13.1: Describe in detail the selection of the individuals included in the study (that is, study population selection) including filtering based on data quality, data availability, and linkage. The selection of included individuals can be described in the text or by means of the study flow diagram. | — | Figure 2 |
| Descriptive data | | | | |
| 14 | (a) Give characteristics of study participants (eg, demographic, clinical, social) and information on exposures and potential confounders.  (b) Indicate the number of participants with missing data for each variable of interest.  (c) Cohort study—summarise follow-up time (eg, average and total amount). | — | — | 11-13, Table 2, Table S19 |
| Outcome data | | | | |
| 15 | Cohort study—report numbers of outcome events or summary measures over time. Case-control study—report numbers in each exposure category, or summary measures of exposure. Cross sectional study—report numbers of outcome events or summary measures. | — | — | 11-13 |
| Main results | | | | |
| 16 | (a) Give unadjusted estimates and, if applicable, confounder adjusted estimates and their precision (eg, 95% confidence intervals). Make clear which confounders were adjusted for and why they were included.  (b) Report category boundaries when continuous variables are categorised.  (c) If relevant, consider translating estimates of relative risk into absolute risk for a meaningful time period. | — | — | 11-13, Figures 3 and 4, Table S18 |
| Other analyses | | | | |
| 17 | Report other analyses done—eg, analyses of subgroups and interactions, and sensitivity analyses. | — | — | 11-13, Figure 3, Table 3, Table S18, Figures 12 and 13, Table S20 |
| **Discussion** | | | | |
| Key results | | | | |
| 18 | Summarise key results with reference to study objectives. | — | — | 13-14 |
| Limitations | | | | |
| 19 | Discuss limitations of the study, taking into account sources of potential bias or imprecision. Discuss both direction and magnitude of any potential bias. | 19.1: Discuss the implications of using data that were not created or collected to answer the specific research question(s). Include discussion of misclassification bias, unmeasured confounding, missing data, and changing eligibility over time, as they pertain to the study being reported. | 19.1.a: Describe the degree to which the chosen database(s) adequately captures the drug exposure(s) of interest. | 17-18 |
| Interpretation | | | | |
| 20 | Give a cautious overall interpretation of results considering objectives, limitations, multiplicity of analyses, results from similar studies, and other relevant evidence. | — | 20.a: Discuss the potential for confounding by indication, contraindication or disease severity or selection bias (healthy adherer/sick stopper) as alternative explanations for the study findings when relevant. | 13-18 |
| Generalisability | | | | |
| 21 | Discuss the generalisability (external validity) of the study results. | — | — | 16-17 |
| **Other information** | | | | |
| Funding | | | | |
| 22 | Give the source of funding and the role of the funders for the present study and, if applicable, for the original study on which the present article is based. | — | — | Private until publication |
| Accessibility of protocol, raw data, and programming code | | | | |
| 22 | — | 22.1: Authors should provide information on how to access any supplemental information such as the study protocol, raw data, or programming code. | — | 7 |

RECORD=reporting of studies conducted using observational routinely collected data; RECORD-PE=RECORD for pharmacoepidemiological research; STROBE=strengthening the reporting of observational studies in epidemiology.

*REFERENCE: Langan SM, Schmidt S, Wing K, Ehrenstein V, Nicholls S, Filion K, Klungel O, Petersen I, Sorensen H, Guttmann A, Harron K, Hemkens L, Moher D, Schneeweiss S, Smeeth L, Sturkenboom M, von Elm E, Wang S, Benchimol EI.  The REporting of studies Conducted using Observational Routinely-collected health Data (RECORD) Statement for Pharmacoepidemiology (RECORD-PE). *BMJ* 2018; 363: k3532.

Figure S5. Examples of dividing person-time into periods for the marginal structural model and proportional hazards model.


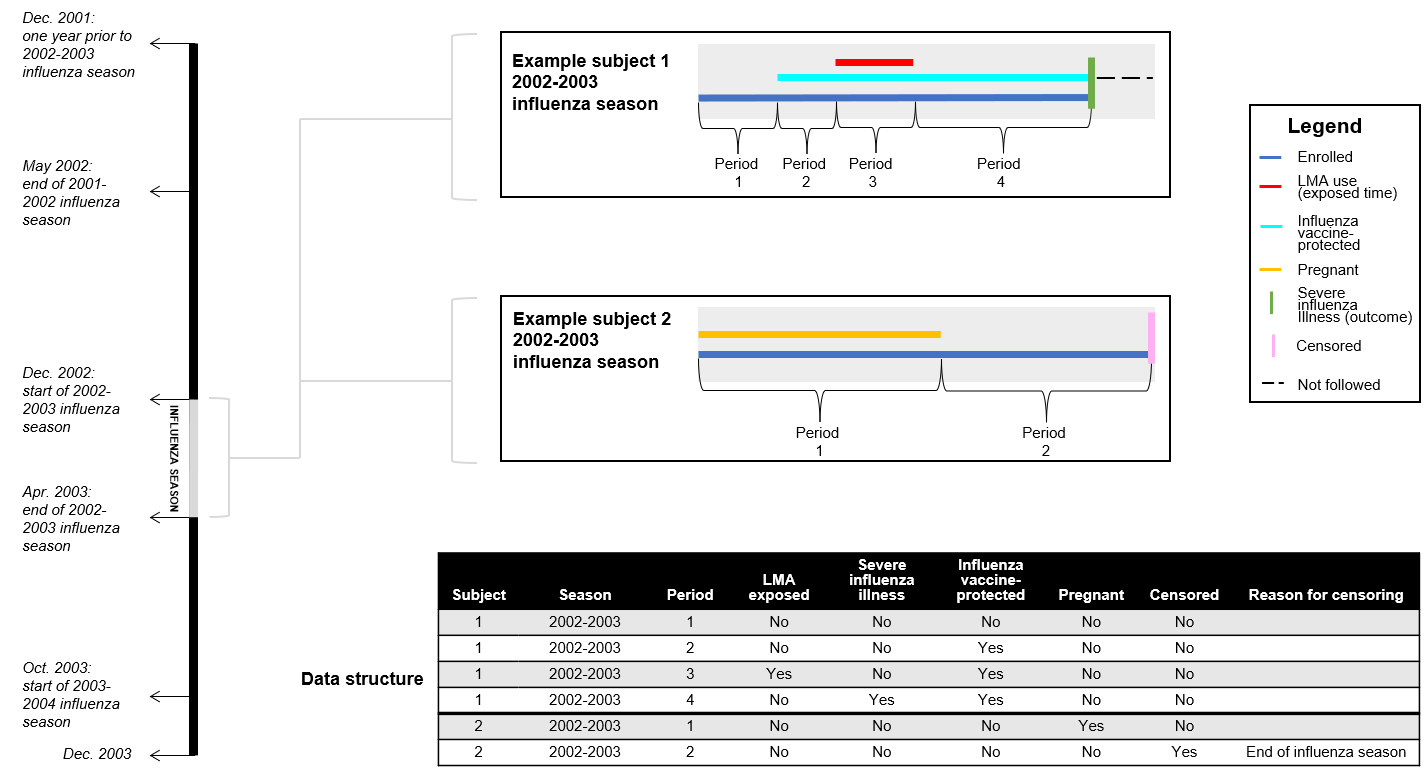


This figure depicts a two-year timeframe during which example subjects were enrolled in TennCare or the DoD MHS. These subjects were continuously enrolled at least one year prior to the 2002-2003 influenza season and had leukotriene modifying agent (LMA) exposure in the year prior to the 2002-2003 influenza season, which were criteria for inclusion in our study population. We restricted analyses to influenza seasons when individuals are at highest risk for severe influenza illness (outcome) and to minimize misclassification. Followed time (i.e., “person time”) is represented by colored, solid horizontal lines. LMA use (i.e., exposed time) is depicted by horizontal red lines. Unexposed time includes followed time during which the individual is not exposed to LMAs (horizontal blue or yellow lines; also labeled as “No” in “LMA exposed” column in data structure table). Time during which subjects are not followed is depicted as horizontal, dashed black lines.

Subject 1 is enrolled at the start of the 2002-2003 influenza season. During the season, they receive the influenza vaccination, which begins a new period as their risk for severe influenza illness decreases. They are then exposed to an LMA, which begins their “exposed time”. Prior to starting the LMA (i.e., during periods 1 and 2) the subject was considered to have been “unexposed”. The end of LMA exposure time (i.e., last day of supply) triggers the end of period 3. During period 4, the subject remains enrolled and influenza-vaccine protected and is considered LMA unexposed. This period ends when the subject has a severe influenza illness. This ends their follow up time for the 2002-2003 season (follow up time can resume at the start of the 2003-2004 if they were continuously enrolled at least one year prior to the 2003-2004 season and had LMA exposure in the year prior to the 2003-2004 season).

Subject 2 is enrolled and pregnant at the start of the 2002-2003 influenza season. During the season, their pregnancy ends, which triggers the end of period 1 as their risk for severe influenza illness decreases. They continue enrollment through the end of the season when they are censored. This subject was never exposed to LMAs during the 2002-2003 influenza season, and is thus, considered “unexposed” throughout the season.

Table S10. Covariate definitions.

| **Covariate** | **Definition** | **Coding** |
| --- | --- | --- |
| Prior LMA coverage | Proportion of days covered (PDC) by LMAs in the 365 days prior to the period start date. PDC was calculated using the following equation [References 1 and 2 in footnote]:  $PDC=\frac{Number of days in period covered}{365 days}$  Where ‘number of days in period covered’ was equal to the number of days of supply (not counting overlapping days; see example).  Example:  30 days of supply  365 days prior to period start  Period start  Influenza season  30 days of supply  5-day overlap  $PDC=\frac{\left( 30+30 \right)-5}{365}= 0.15$ | Continuous (range 0-1) |
| Sex | - Self-reported sex defined at enrollment (TennCare) - Information on self-reported sex may have been captured multiple times in the DoD MHS. We used the following algorithm to define sex for individuals with sex that differed across periods (0.1% individuals):  1. Use sex that appears most often across periods 2. For ties, use first recorded sex | Male, female |
| Race and  ethnicity | - Self-reported race and ethnicity defined at enrollment (TennCare). TennCare provided race and ethnicity categories. Data used for race and ethnicity come from a third-party data processor that creates mutually exclusive categories of race and ethnicity. This grouping may not reflect other sources of Medicaid data in Tennessee (like T-MSIS). - As self-reported race and ethnicity were missing for 63% of DoD MHS population, race and ethnicity were not included in the analyses. | American Indian, Asian, Black, Hispanic/Latin,  Other/unknown, Southeast Asian,  White |
| Asthma and/or allergic rhinitis status | Asthma and/or allergic rhinitis status at any time prior to the period start date. Coding for this variable may change over time if an individual developed another condition over the study period (e.g., asthma only to both asthma and allergic rhinitis). | Asthma only, allergic rhinitis only, both asthma and allergic rhinitis |
| Acute asthma healthcare utilization | Hierarchical acute asthma healthcare utilization in the 365 days prior the period start date. If more than one asthma encounter occurred in this time frame, the most severe encounter was coded.   - One or more oral corticosteroid fills (national drug codes listed in Table S11 [see Supplementary Data excel file]) during this time frame fulfilled this definition. - Asthma-related emergency department visit definition:   - A primary (i.e., in the first position) ICD-9 code diagnosis of 493.xx (asthma) or ICD-10 code J45.xx (asthma) during the emergency department visit OR   - A secondary (i.e., in any position after the primary diagnosis) ICD-9 diagnosis of 493.xx (asthma) or ICD-10 code J45.xx (asthma) during the emergency department visit in addition to one of the following primary respiratory diagnoses during the same emergency department visit:     - Pneumonia (ICD-9 codes 480.x-486; ICD-10 codes J12.xx-J18.x)     - Influenza with pneumonia or other respiratory manifestations (ICD-9 codes 487.0, 487.1; ICD-10 codes J09.xx-J11.xx)     - Bronchitis (ICD-9 code 490; ICD-10 code J40)     - Acute upper respiratory infection (ICD-9 code 465.x; ICD-10 code J06.x)     - Acute bronchitis and bronchiolitis (ICD-9 code 466.xx; ICD-10 codes J20.x, J21.x)     - Pulmonary collapse (ICD-9 code 518.0; ICD-10 codes J98.11, J98.19)     - Respiratory failure (ICD-9 code 518.81; ICD-10 codes J96.00, J96.90)     - Other pulmonary insufficiency (ICD-9 code 518.82; ICD-10 code J80)     - Pneumothorax (ICD-9 code 512.xx; ICD-10 code J93.xx)     - Perinatal chronic respiratory disease (ICD-9 code 770.7; ICD-10 codes P27.x)     - Viral infections, excluding human papillomavirus, retrovirus, retrovirus NOS, HTLV I, HTLV II, HIV 2, retrovirus NEC (ICD-9 codes 079.0, 079.1, 079.2, 079.3, 079.6, 079.8, 079.9; ICD-10 codes B97.0, B97.1x, B97.2, B97.4, B97.5, B97.6, B97.8) - Asthma-related 23-hour/observational stay definition:   - A primary (i.e., in the first position) ICD-9 code diagnosis of 493.xx (asthma) or ICD-10 code J45.xx (asthma) during the 23-hour/observational stay OR   - A secondary (i.e., in any position after the primary diagnosis) ICD-9 diagnosis of 493.xx (asthma) or ICD-10 code J45.xx (asthma) during the 23-hour/observational stay in addition to one of the following primary respiratory diagnoses during the same 23-hour/observational visit:     - Pneumonia (ICD-9 codes 480.x-486; ICD-10 codes J12.xx-J18.x)     - Influenza with pneumonia or other respiratory manifestations (ICD-9 codes 487.0, 487.1; ICD-10 codes J09.xx-J11.xx)     - Bronchitis (ICD-9 code 490; ICD-10 code J40)     - Acute upper respiratory infection (ICD-9 code 465.x; ICD-10 code J06.x)     - Acute bronchitis and bronchiolitis (ICD-9 code 466.xx; ICD-10 codes J20.x, J21.x)     - Pulmonary collapse (ICD-9 code 518.0; ICD-10 codes J98.11, J98.19)     - Respiratory failure (ICD-9 code 518.81; ICD-10 codes J96.00, J96.90)     - Other pulmonary insufficiency (ICD-9 code 518.82; ICD-10 code J80)     - Pneumothorax (ICD-9 code 512.xx; ICD-10 code J93.xx)     - Perinatal chronic respiratory disease (ICD-9 code 770.7; ICD-10 codes P27.x)     - Viral infections, excluding human papillomavirus, retrovirus, retrovirus NOS, HTLV I, HTLV II, HIV 2, retrovirus NEC (ICD-9 codes 079.0, 079.1, 079.2, 079.3, 079.6, 079.8, 079.9; ICD-10 codes B97.0, B97.1x, B97.2, B97.4, B97.5, B97.6, B97.8) - Asthma-related hospitalization definition:   - A primary (i.e., in the first position) ICD-9 code diagnosis of 493.xx (asthma) or ICD-10 code J45.xx (asthma) during the hospitalization OR   - A secondary (i.e., in any position after the primary diagnosis) ICD-9 diagnosis of 493.xx (asthma) or ICD-10 code J45.xx (asthma) during the hospitalization in addition to one of the following primary respiratory diagnoses during the same hospitalization:     - Pneumonia (ICD-9 codes 480.x-486; ICD-10 codes J12.xx-J18.x)     - Influenza with pneumonia or other respiratory manifestations (ICD-9 codes 487.0, 487.1; ICD-10 codes J09.xx-J11.xx)     - Bronchitis (ICD-9 code 490; ICD-10 code J40)     - Acute upper respiratory infection (ICD-9 code 465.x; ICD-10 code J06.x)     - Acute bronchitis and bronchiolitis (ICD-9 code 466.xx; ICD-10 codes J20.x, J21.x)     - Pulmonary collapse (ICD-9 code 518.0; ICD-10 codes J98.11, J98.19)     - Respiratory failure (ICD-9 code 518.81; ICD-10 codes J96.00, J96.90)     - Other pulmonary insufficiency (ICD-9 code 518.82; ICD-10 code J80)     - Pneumothorax (ICD-9 code 512.xx; ICD-10 code J93.xx)     - Perinatal chronic respiratory disease (ICD-9 code 770.7; ICD-10 codes P27.x)     - Viral infections, excluding human papillomavirus, retrovirus, retrovirus NOS, HTLV I, HTLV II, HIV 2, retrovirus NEC (ICD-9 codes 079.0, 079.1, 079.2, 079.3, 079.6, 079.8, 079.9; ICD-10 codes B97.0, B97.1x, B97.2, B97.4, B97.5, B97.6, B97.8) | No encounter (least severe), oral corticosteroid fill, asthma-related emergency department visit, asthma-related 23-hour [TennCare]/  observational [DoD MHS] visit, asthma-related hospitalization (most severe) |
| Number of asthma encounters | Sum of oral corticosteroid fills, asthma-related emergency department visits, asthma-related 23-hour/observational stays, and hospitalizations during the 365 days prior to the period start date. | Continuous |
| Asthma controller medication coverage | Proportion of days covered (PDC) by non-LMA asthma controller medications (including inhaled corticosteroids, inhaled corticosteroid/long-acting beta-agonists, long-acting beta-agonists, oral mast cell stabilizers, methylxanthines, anticholinergic/short-acting beta-agonist combinations, antibody inhibitors, and anticholinergics) in the 365 days prior to the period start date. PDC was calculated using the following equation [References 1 and 2 in footnote]:  $PDC=\frac{Number of days in period covered}{365 days}$  Where ‘number of days in period covered’ was equal to the number of days of supply (not counting overlapping days). | Continuous (range 0-1) |
| Allergic rhinitis medication coverage | Proportion of days covered (PDC) by non-LMA allergic rhinitis medications (including oral antihistamine and antihistamine-decongestant combinations, NCS, nasal mast cell stabilizers, nasal antihistamines, ophthalmic antihistamines, and ophthalmic mast cell stabilizers) in the 365 days prior to the period start date. PDC was calculated using the following equation [References 1 and 2 in footnote]:  $PDC=\frac{Number of days in period covered}{365 days}$  Where ‘number of days in period covered’ was equal to the number of days of supply (not counting overlapping days). | Continuous (range 0-1) |
| Number of asthma rescue medication fills | Number of short-acting beta-agonist (SABA) fills in the 365 days prior to the period start date. | Continuous |
| Age at period start date | Age at period start date (rounded to nearest year)   - Calculation:   $\frac{Period start date-date of birth}{365.25}$ | Continuous |
| Age at start of influenza season | Age at the start of the respective influenza season (rounded to nearest year)   - Calculation:   $\frac{Season start date-date of birth}{365.25}$ | Continuous |
| Charlson Comorbidity Index | Charlson Comorbidity Index at period start date. Individuals were considered to have a comorbidity if they had one or more of the ICD codes listed for each comorbidity (Table S12) at any time prior to the period start date. | Continuous |
| Antibiotic use | Antibiotic use in the 365 days period to the period start date. Antibiotic use was defined by having one or more fill with the national drug codes listed in Table S13. | Yes, no |
| Tobacco use | Tobacco use in the 365 days prior to the period start date.   - Tobacco use was defined by having one or more of the following:   - Self-reported tobacco use (only available in DoD MHS)   - ICD-9 codes 305.1, V15.82, 649.0x, 989.84; ICD-10 codes F17.xxx, Z87.891, Z72.0, O99.33x, Z71.6, T65.2xxxx   - CPT® codes 99406, 99407, S9075, S9453, G0375, G0376, G0436, G0437, G8402, G8403, G8453, G8454, S4990, S4991, S4995, 4000F, 4001F, 4004F, G9016, G9276, G9458, 1032F, 1034F   - Prescription nicotine replacement therapy tobacco cessation medications (generic names: varenicline tablets, nicotine inhaler, nicotine spray pump) (Table S14) | Yes, no |
| Influenza vaccination | Influenza vaccine-protected in the current period.   - Influenza vaccination was defined by having one or more of the following:   - CPT® codes 90630, 90653-90658, 90660, 90662, 90672-90674, 90682, 90685-90689, 90694, 90756, Q2034-Q2039, G0008   - Any national drug code listed in Table S15   Vaccine protection was considered to begin 14 days after vaccine receipt. If one or more of the above listed codes were identified during a period, all subsequent periods within the respective influenza season were defined as vaccine protected (i.e., once influenza vaccination was established, this coding remained for the entirety of that specific influenza season to capture the protected effect of the individual from severe influenza illness). | Yes, no |
| Prior influenza vaccination | Influenza vaccination in the 365 days prior to the period start date. Influenza vaccination was defined in the previous row. | Yes, no |
| Neuraminidase inhibitor use | Neuraminidase inhibitor use in the 365 days prior to the period start date. Neuraminidase inhibitors use was defined by having one or more fill with the national drug codes listed in Table S16 (see Supplementary Data excel file). | Yes, no |
| Nursing home residence | Nursing home residence in the 365 days prior to the period start date.   - Nursing home residence was defined by having one or more of the following:   - CPT® codes 99304-99306, 99307-99310, 99315-99316, 99318 | Yes, no |
| Pregnancy | Pregnant in the current period. Women must have met the below delivery or miscarriage/abortion definitions from ages 15-50 years to be defined as pregnant.   - End of pregnancy was defined as date of delivery, miscarriage, or abortion.   - Delivery was defined by having one or more of the following:     - CPT® codes 59409, 59410, 59514, 59515, 59612, 59614, 59621, 59622     - ICD-9 codes 650-659, 660-669, V27.x, V30-V39     - ICD-10 codes O60-O77, O80-O82, Z37.xx, Z38.xx - Miscarriage and/or abortion was defined by having one or more of the following:   - ICD-9 codes 632, 633.xx, 634.xx, 635.xx, 636.xx, 637.xx, 639.x   - ICD-10 codes O00.xx, O02.1, O03.x, Z33.2, or O04.xx   - CPT® codes 59120, 59121, 59130, 59135, 59136, 59140, 59150, 59151, 59820, 59821, 59830, 59840, 59841, 59850, 59851, 59852, 59855, 59856, 59857   Determination of pregnancy start date:   - For women with one or more gestational age codes (listed below) in addition to delivery date or miscarriage/abortion date information (ideal situation), the latest gestational age code available was used to determine the woman’s pregnancy start date.   - Example: If a woman’s latest ICD-9 code related to gestational age is 765.25 (29-30 weeks of gestation), then we counted back 29.5 weeks to estimate the pregnancy start date. - For women with only one gestational age code and that gestational code was ICD-9 765.21 (<24 weeks), then we counted back 23 weeks to estimate the pregnancy start date. - For women with only one gestational age code and that gestational code was ICD-9 765.29 (≥37 weeks), we counted back 39 weeks to estimate the pregnancy start date. - For women with only delivery date available (i.e., missing gestational age code and no miscarriage/abortion date), we counted back 39 weeks (9 months) to estimate the pregnancy state date. - For women with only miscarriage/abortion date available (i.e., missing gestational age code and no delivery date), we counted back 13 weeks (3 months) to estimate the pregnancy start date. - If multiple delivery dates or miscarriage/abortion dates occurred within ≤90 days, only the first delivery date or miscarriage/abortion date was counted to help differentiate between multiple pregnancies per woman. - Gestational age as having one or more of the following:   - ICD-9 code 765.0x, 765.1x, 765.21-765.29   - ICD-10 code Z3A.01-Z3A.09, Z3A.1x, Z3A.2x, Z3A.3x, Z3A.4x | Yes, no |
| Influenza season | Influenza season during which the period occurs | 1997-1998 [TennCare only], 1999 [TennCare only],  1999-2000 [TennCare only], 2000-2001 [TennCare only], 2001-2002 [TennCare only], 2002-2003 [TennCare only], 2003-2004, 2005 [TennCare only], 2004-2005 [DoD MHS only],  2005-2006, 2006-2007, 2007-2008, 2008-2010 [TennCare only], 2009-2010 [DoD MHS only], 2010-2011, 2012,  2012-2013, 2013-2014, 2014-2015, 2016,  2016-2017, 2017-2018, 2018-2019 [TennCare only], 2019-2020 [TennCare only] |

LMA, leukotriene modifying agent.

[1] Benner JS, Glynn RJ, Mogun H, Neumann PJ, Weinstein MC, Avorn J. Long-term Persistence in Use of Statin Therapy in Elderly Patients. JAMA. 2002;288(4):455-61. doi: 10.1001/jama.288.4.455.

[2] Prieto-Merino D, Mulick A, Armstrong C, Hoult H, Fawcett S, Eliasson L, et al. Estimating proportion of days covered (PDC) using real-world online medicine suppliers’ datasets. Journal of Pharmaceutical Policy and Practice. 2021;14(1):113. doi: 10.1186/s40545-021-00385-w.

Table S11 is provided in the Supplementary Data excel file**.**

Table S12. Charlson Comorbidity Index categories and associated diagnosis codes and points.

| **Comorbidity** | **ICD-9/10 codes** | **Point(s)** |
| --- | --- | --- |
| Myocardial infarction | 410.x, 412.x, I21.x, I22.x, I25.2 | 1 |
| Congestive heart failure | 398.91, 402.01, 402.11, 402.91, 404.01, 404.03, 404.11, 404.13, 404.91, 404.93, 425.4, 425.5, 425.6, 425.7, 425.8, 425.9, 428.x, I11.0, I13.0, I13.2, I25.5, I42.0, I42.5, I42.6, I42.7, I42.8, I42.9, I43.x, I50.x, P29.0 | 1 |
| Peripheral vascular disease | 093.0, 437.3, 440.x, 441.x, 443.1, 443.2x, 443.8x, 443.9, 447.1, 557.1, 557.9, V43.4, I70.x, I71.x, I73.1, I73.8, I73.9, I77.1, I79.0, I79.1, I79.8, K55.1, K55.8, K55.9, Z95.8, Z95.9 | 1 |
| Cerebrovascular disease | 362.34, 430.x, 431.x, 432.x, 433.x, 434.x, 435.x, 436.x, 437.x, 438.x, G45.x, G46.x, H34.0x, H34.1x, H34.2x, I60.x, I61.x, I62.x, I63.x, I64.x, I65.x, I66.x, I67.x, I68.x | 1 |
| Dementia | 290.0, 290.1x, 290.2x, 290.3, 290.4x, 294.0, 294.1x, 294.2x, 294.8, 331.0, 331.1x, 331.2, 331.7, 797, F01.x, F02.x, F03.x, F04, F05, F06.1, F06.8, G13.2, G13.8, G30.x, G31.0x, G31.1, G31.2, G91.4, G94, R41.81, R54 | 1 |
| Chronic pulmonary disease | 490.x, 491.x, 492.x, 493.x, 494.x, 495.x, 496.x, 500.x, 501.x, 502.x, 503.x, 504.x, 505.x, 506.4, 508.1, 508.8, J40.x, J41.x, J42.x, J43.x, J44.x, J45.x, J46.x, J47.x, J60.x, J61.x, J62.x, J63.x, J64.x, J65.x, J66.x, J67.x, J68.4, J70.1, J70.3 | 1 |
| Rheumatic disease | 446.5, 710.0, 710.1, 710.2, 710.3, 710.4, 714.0, 714.1, 714.2, 714.8x, 725.x, M05.x, M06.x, M31.5, M32.x, M33.x, M34.x, M35.1, M35.3, M36.0 | 1 |
| Peptic ulcer disease | 531.x, 532.x, 533.x, 534.x, K25.x, K26.x, K27.x, K28.x | 1 |
| Mild liver disease | 070.22, 070.23, 070.32, 070.33, 070.44, 070.54, 070.6, 070.9, 570.x, 571.x, 573.3, 573.4, 573.8, 573.9, V42.7, B18.x, K70.0, K70.1, K70.2, K70.3, K70.9, K71.3, K71.4, K71.5, K71.7, K73.x, K74.x, K76.0, K76.2, K76.3, K76.4, K76.8, K76.9, Z94.4 | 1 |
| Diabetes without chronic complications | 250.8x, 250.9x, 249.0x, 249.1x, 249.2x, 249.3x, 249.9x, E08.0x, E08.1x, E08.6x, E08.8x, E08.9x, E09.0x, E09.1x, E09.6x, E09.8x, E09.9x, E10.1x, E10.6x, E10.8x, E10.9x, E11.0x, E11.1x, E11.6x, E11.8x, E11.9x, E13.0x, E13.1x, E13.6x, E13.8x, E13.9x | 1 |
| Mild to moderate renal disease | 403.00, 403.10, 403.90, 404.00, 404.01, 404.10, 404.11, 404.90, 404.91, 582.x, 583.x, 585.1, 585.2, 585.3, 585.4, 585.9, V42.0, I12.9, I13.0, I13.10, N03.x, N05.x, N18.1, N18.2, N18.3, N18.4, N18.9, Z94.0 | 1 |
| Diabetes with chronic complications | 250.4, 250.5, 250.6, 250.7, E08.2, E08.3, E08.4, E08.5, E09.2, E09.3, E09.4, E09.5, E10.2, E10.3, E10.4, E10.5, E11.2, E11.3, E11.4, E11.5, E13.2, E13.3, E13.4, E13.5 | 2 |
| Hemiplegia or paraplegia | 334.1, 342.x, 343.x, 344.x, G04.1, G11.4, G80.0, G80.1, G80.2, G81.x, G82.x, G83.x | 2 |
| Malignancy | 14x.x, 15x.x, 16x.x, 170.x, 171.x, 172.x, 174.x, 175.x, 176.x, 179.x, 18x.x, 190.x, 191.x, 192.x, 193.x, 194.x, 195.x, 199.1, 200.x, 201.x, 202.x, 203.x, 204.x, 205.x, 206.x, 207.x, 208.x, 238.6, C0x.x, C1x.x, C2x.x, C30.x, C31.x, C32.x, C33.x, C34.x, C37.x, C38.x, C39.x, C40.x, C41.x, C43.x, C45.x, C46.x, C47.x, C48.x, C49.x, C50, C51-58.x, C60-63.x, C76.x, C80.1, C81.x, C82.x, C83.x, C84.x, C85.x, C88.x, C9x.x | 2 |
| **Comorbidity** | **ICD-9/10 codes** | **Point(s)** |
| Moderate to severe liver disease | 456.0, 456.1, 456.2x, 572.2, 572.3, 572.4, 572.8, I85.0x, I86.4, K70.4x, K71.1x, K72.1x, K72.9x, K76.5, K76.6, K76.7 | 3 |
| Severe renal disease | 403.01, 403.11. 403.91, 404.02, 404.03, 404.12, 404.13, 404.92, 404.93, 585.5, 585.6, 586.x, 588.0, V45.11, V45.12, V56.0, V56.1, V56.2, V56.31, V56.32, V56.8, I12.0, I13.11, I13.2, N18.5, N18.6, N19.x, N25.0, Z49.x, Z99.2 | 3 |
| HIV infection, without AIDS | 042.x, B20.x | 3 |
| Metastatic solid tumor | 196.x, 197.x, 198.x, 199.0, C77.x, C78.x, C79.x, C80.0, C80.2 | 6 |
| AIDS (HIV infection + opportunistic infection)^a^ | HIV infection: 042.x, B20.x  Opportunistic infection: 112.x, 180.x, 114.x, 117.5, 007.4, 078.5, 348.3x, 054.x, 115.x, 007.2, 176.x, 200-209, 031.x, 010-018, 136.3, V12.61, 046.3, 003.1, 130.x, 799.4, B37.x, C53.x, B38.x, B45.x, A07.2, B25.x, G93.4x, B00, B39.x, A07.3, C46.x, C81-C96, A31.x, A15-A19, B59, Z87.01, A81.2, A02.1, B58.x, R64 | 6 |

^a^Subjects identified as having AIDS if they had at least one ICD-9/10 code for HIV at any time prior to or during the study period AND they had at least one ICD-9/10 code for an opportunistic infection during the study period.

Reference: Glasheen WP, Cordier T, Gumpina R, Haugh G, Davis J, Renda A. Charlson comorbidity index: ICD-9 update and ICD-10 translation. *Am Health Drug Benefits*, 2019. 12(4): 188-197.

Tables S13-16 are provided in the Supplementary Data excel file**.**

Table S17. Chronic obstructive pulmonary disease (COPD) definition used in secondary analysis for the marginal structural model.

| **Definition** |
| --- |
| COPD at period start date. Individuals were considered to have COPD if they were ≥35 years at time of diagnosis and had one or more of the following ICD codes listed at any time prior to the period start date:  ICD-9 code 490 (bronchitis, not specified as acute or chronic)  ICD-9 code 491.xx (chronic bronchitis)  ICD-9 code 492.x (emphysema)  ICD-9 code 494.x (bronchiectasis)  ICD-9 code 495.x (extrinsic allergic alveolitis)  ICD-9 code 496 (chronic airway obstruction, not elsewhere classified)  ICD-10 code J40 (bronchitis, not specified as acute or chronic)  ICD-10 code J41.x (simple and mucopurulent chronic bronchitis)  ICD-10 code J42 (unspecified chronic bronchitis)  ICD-10 code J43.x (emphysema)  ICD-10 code J44.x (other chronic obstructive pulmonary disease)  ICD-10 code J47.x (bronchiectasis) |

Table S18. Results from marginal structural model analyses by population and meta-analyzed.

| **Analysis** | **TennCare** | | | | | | | | | | |
| --- | --- | --- | --- | --- | --- | --- | --- | --- | --- | --- | --- |
|  | **Sample size (# individuals/ # periods)** | **No LMA** | | | **LMA** | | | **IRR**  **(95% CI)** | **aIRR**  **(95% CI)** | **Adjusted p-value** |  |
|  |  | **Severe influenza illness (periods)** | **Person-days** | **Incidence rate/100,000 days** | **Severe influenza illness**  **(periods)** | **Person-days** | **Incidence rate/100,000 days** |  |  |  |  |
| Primary | 363,694/  2,340,952 | 292 | 120,855,406 | 0.24 | 167 | 40,758,984 | 0.41 | 1.70  (1.40, 2.05) | 1.26  (0.99, 1.59) | 0.06 |  |
| Montelu-  kast only | 363,541/  2,327,941 | 292 | 120,855,406 | 0.24 | 156 | 40,048,755 | 0.39 | 1.61  (1.33, 1.96) | 1.27  (1.00, 1.61) | 0.05 |  |
| Secondary |  |  |  |  |  |  |  |  |  |  |  |
| COPD  subgroup | 49,236/  231,092 | 97 | 9,304,760 | 1.04 | 88 | 5,892,865 | 1.49 | 1.43  (1.07, 1.91) | 1.06  (0.76, 1.47) | 0.73 |  |
| <5 years  subgroup | 78,089/  263,304 | 39 | 14,606,967 | 0.27 | 11 | 3,986,940 | 0.28 | 1.03  (0.53, 2.02) | 1.45  (0.69, 3.04) | 0.33 |  |
| ≥65 years  subgroup | 9,473/  33,568 | 18 | 725,837 | 2.48 | 17 | 1,028,208 | 1.65 | 0.67  (0.34, 1.29) | 0.54  (0.26, 1.13) | 0.10 |  |
| Allergic  rhinitis only  subgroup | 120,601/  444,722 | 15 | 30,464,336 | 0.05 | 5 | 6,155,330 | 0.08 | 1.65  (0.60, 4.54) | 2.13  (0.73, 6.22) | 0.17 |  |
| Pregnant  subgroup | 2,430/  3,997 | 6 | 249,277 | 2.41 | 0 | 30,001 | 0 | -- | -- | -- |  |
| **Analysis** | **DoD MHS** | | | | | | | | | | |
|  | **Sample size (# individuals/ # periods)** | **No LMA** | | | **LMA** | | | **IRR**  **(95% CI)** | **aIRR**  **(95% CI)** | **Adjusted p-value** |  |
|  |  | **Severe influenza illness**  **(periods)** | **Person-days** | **Incidence rate/100,000 days** | **Severe influenza illness**  **(periods)** | **Person-days** | **Incidence rate/100,000 days** |  |  |  |  |
| Primary | 711,883/  4,201,265 | 331 | 221,853,849 | 0.15 | 279 | 125,177,318 | 0.22 | 1.49  (1.27, 1.75) | 1.01  (0.84, 1.21) | 0.89 |  |
| Montelu-  kast only | 711,823/  4,193,853 | 331 | 221,853,849 | 0.15 | 279 | 124,674,798 | 0.22 | 1.50  (1.28, 1.76) | 1.01  (0.85, 1.21) | 0.89 |  |
| Secondary |  |  |  |  |  |  |  |  |  |  |  |
| COPD  subgroup | 106,430/  666,032 | 105 | 28,063,486 | 0.37 | 119 | 26,560,961 | 0.45 | 1.20  (0.92, 1.56) | 1.01  (0.76, 1.33) | 0.94 |  |
| <5 years  subgroup | 74,367/  206,892 | 25 | 12,166,903 | 0.21 | -- | -- | 0.18 | 0.87  (0.39, 1.92) | 0.85  (0.36, 2.01) | 0.72 |  |
| ≥65 years  subgroup | 18,924/  122,203 | 43 | 4,144,143 | 1.04 | 62 | 5,761,859 | 1.08 | 1.04  (0.70, 1.53) | 0.92  (0.58, 1.44) | 0.71 |  |
| Allergic  rhinitis only  subgroup | 295,846/  1,201,189 | 40 | 78,681,844 | 0.05 | 24 | 32,688,492 | 0.07 | 1.44  (0.87, 2.40) | 0.80  (0.46, 1.41) | 0.45 |  |
| Pregnant  subgroup | 21,108/  38,877 | -- | -- | 0.24 | -- | -- | 0.46 | 1.95  (0.47, 8.17) | 1.33  (0.33, 5.41) | 0.69 |  |
| **Analysis** | **TennCare and DoD MHS meta-analyzed** | | | | | | | | | |  |
|  | **Sample size (# individuals/ # periods)** | **No LMA** | | | **LMA** | | | **IRR**  **(95% CI)** | **aIRR**  **(95% CI)** | **Adjusted p-value** |  |
|  |  | **Severe influenza illness (periods)** | **Person-days** | **Incidence rate/100,000 days** | **Severe influenza illness (periods)** | **Person-days** | **Incidence rate/100,000 days** |  |  |  |  |
| Primary | 1,075,577/  6,542,217 | 623 | 342,709,255 | 0.18 | 446 | 165,936,302 | 0.27 | 1.57  (1.39, 1.78) | 1.10  (0.95, 1.27) | 0.21 |  |
| Montelu-  kast only | 1,075,364/  6,521,794 | 623 | 342,709,255 | 0.18 | 435 | 164,723,553 | 0.26 | 1.54  (1.36, 1.75) | 1.10  (0.95, 1.26) | 0.21 |  |
| Secondary |  |  |  |  |  |  |  |  |  |  |  |
| COPD  subgroup | 155,666/  897,124 | 202 | 37,368,246 | 0.54 | 207 | 32,453,826 | 0.64 | 1.30  (1.07, 1.58) | 1.03  (0.83, 1.28) | 0.78 |  |
| <5 years  subgroup | 152,456/  470,196 | 64 | 26,773,870 | 0.24 | -- | -- | 0.22 | 0.96  (0.58, 1.60) | 1.15  (0.66, 2.02) | 0.62 |  |
| ≥65 years  subgroup | 28.397/  155,771 | 61 | 4,869,980 | 1.25 | 79 | 6,790,067 | 1.16 | 0.93  (0.66, 1.30) | 0.79  (0.54, 1.17) | 0.24 |  |
| Allergic  rhinitis only  subgroup | 416,447/  1,645,911 | 55 | 109,146,180 | 0.05 | 29 | 38,843,822 | 0.07 | 1.48  (0.94, 2.33) | 0.99  (0.60, 1.62) | 0.96 |  |
| Pregnant  subgroup | 23,538/  42,874 | -- | -- | 0.47 | -- | -- | 0.44 | -- | -- | -- |  |

LMA, leukotriene modifying agent; IRR, incidence rate ratio; aIRR, adjusted incidence rate ratio; CI, confidence interval; COPD, chronic obstructive pulmonary disease.

--, n<5 (TennCare) or n<11 (DoD MHS) severe influenza events. Could not calculate IRR, aIRR, and adjusted p-value due to zero cell counts.

Incidence rate ratios were estimated using Poisson regression. Adjusted incidence rate ratios and corresponding p-values were estimated using a marginal structural model with inverse probability of treatment and censoring weights. For the primary, montelukast only, and COPD subgroup analyses, weights were calculated adjusting for prior LMA coverage, acute asthma healthcare utilization, number of asthma healthcare encounters, asthma controller medication coverage, allergic rhinitis medication coverage, number of asthma rescue medication fills, age at the start of the current period, influenza season, Charlson Comorbidity Index, antibiotic use, tobacco use, influenza vaccination in the current period, prior influenza vaccination, neuraminidase inhibitor use, nursing home residence, asthma and/or allergic rhinitis status, pregnancy, sex, race and ethnicity (race and ethnicity were included for TennCare only), and age during the first period of the respective influenza season. Prior LMA coverage, asthma controller medication coverage, allergic rhinitis medication coverage, age, and Charlson Comorbidity Index were fit with restricted cubic splines For the ≥65 years subgroup analysis, weights were calculated adjusting for the same covariates as the primary analysis, excluding pregnancy. For the <5 years subgroup analysis, weights were calculated adjusting for the same covariates as the primary analysis, excluding pregnancy and nursing home residence. Charlson Comorbidity Index was categorized (0, 1, 2, 3+) and age was adjusted linearly. For the pregnancy subgroup analysis (DoD MHS only), weights were calculated adjusting for the same covariates as the primary analysis, excluding pregnancy. Charlson Comorbidity Index was categorized (0, 1, 2, 3+). For the allergic rhinitis only subgroup analysis, weights were calculated adjusting for the same covariates as the primary analysis, excluding asthma and/or allergic rhinitis status and asthma controller medication coverage (TennCare only). Number of asthma healthcare encounters was categorized (0, 1, 2, 3+). Number of asthma rescue medication fills was categorized (0, 1, 2, 3+) for the analysis among the DoD MHS population and adjusted linearly for the analysis among the TennCare population. Charlson Comorbidity Index was categorized (0, 1, 2, 3+) for the analysis among the TennCare population. Poisson models for all analyses were adjusted for age during the first period in the respective influenza season, sex, and race and ethnicity (race and ethnicity were included for TennCare only).

Figure S6. Example of further dividing person-time by risk of influenza illness for the proportional hazards model.


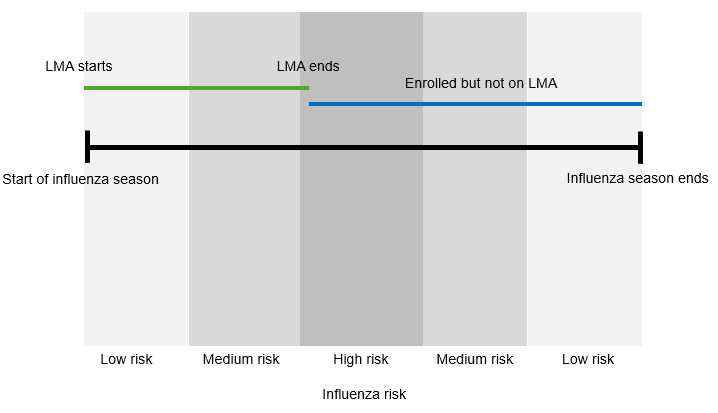


LMA, leukotriene modifying agent. The green horizontal line represents time when the individual is exposed to LMAs. The blue horizontal line represents time when the individual is not exposed to LMAs but are still enrolled in TennCare or the DoD MHS. The underlying influenza risk is illustrated by the shade of gray of the background.

Figure S7. Love plot for comparison of the extent of covariate imbalance in A) TennCare and B) DoD MHS populations using unweighted (crude) comparisons and overlap weights.


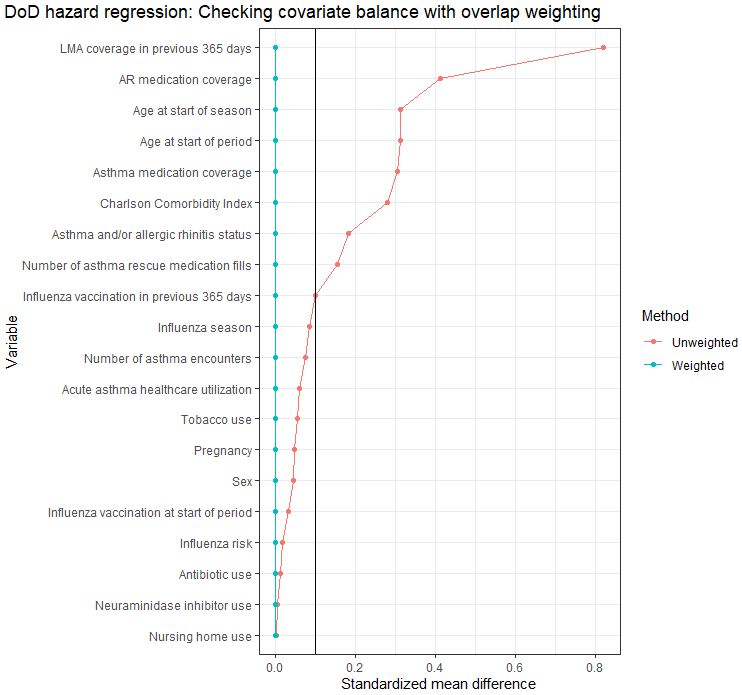

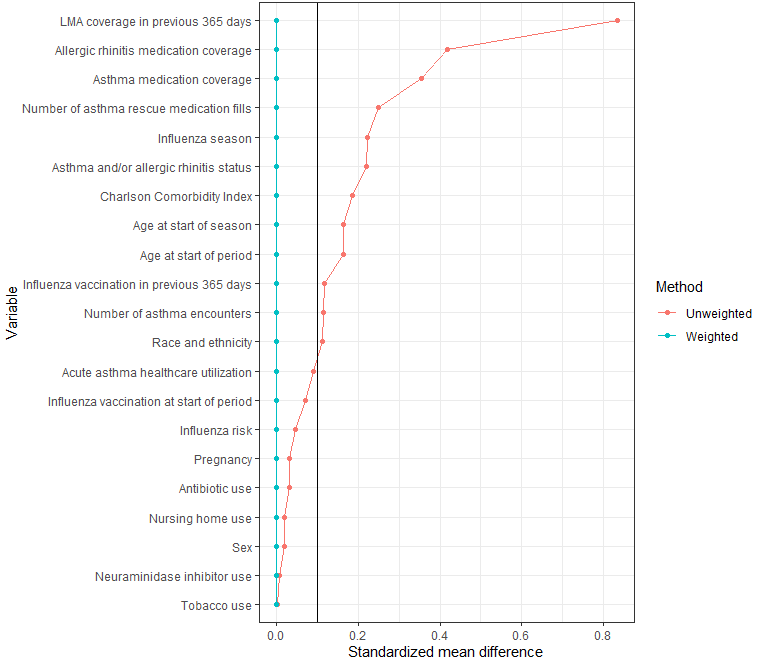


B

A

Figure S8. Distribution of leukotriene modifying agent (LMA) adherence proportion of days covered (PDC) for the Tennessee Medicaid (TennCare) and Department of Defense Military Health System (DoD MHS) populations included in the case-time-control design.


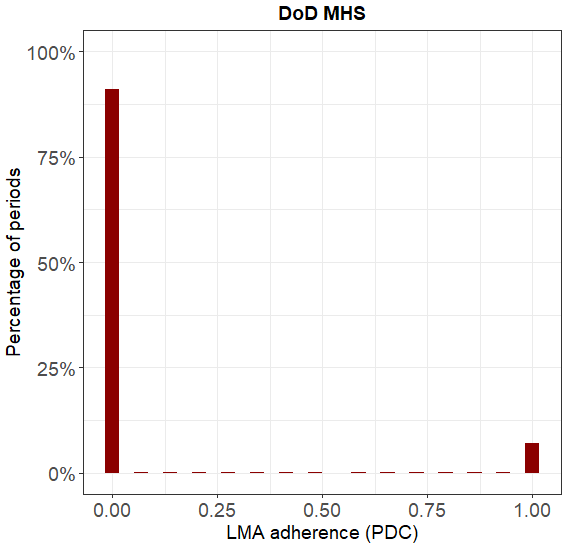

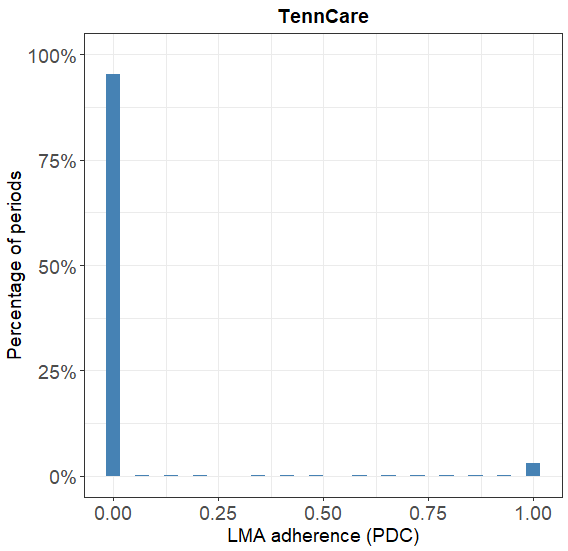


PDC was calculated as the number of days on LMAs during the 14-day case or control and reference periods.


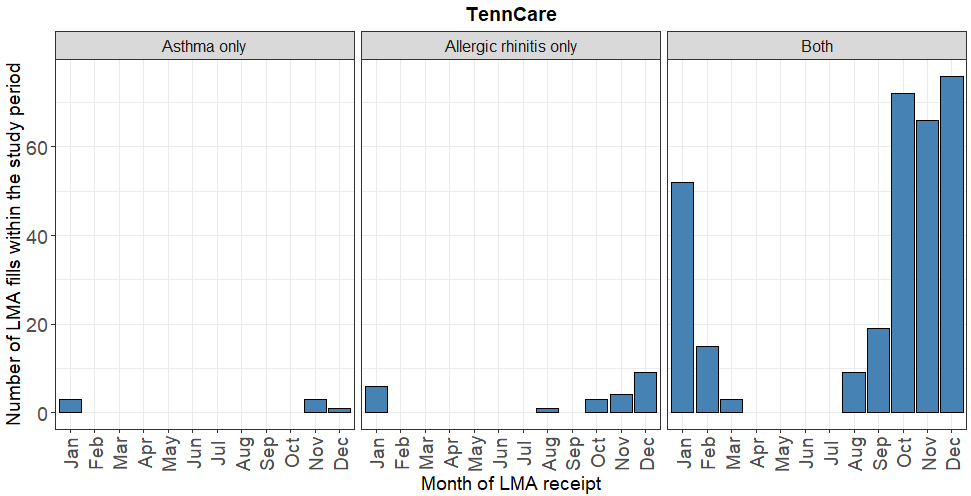

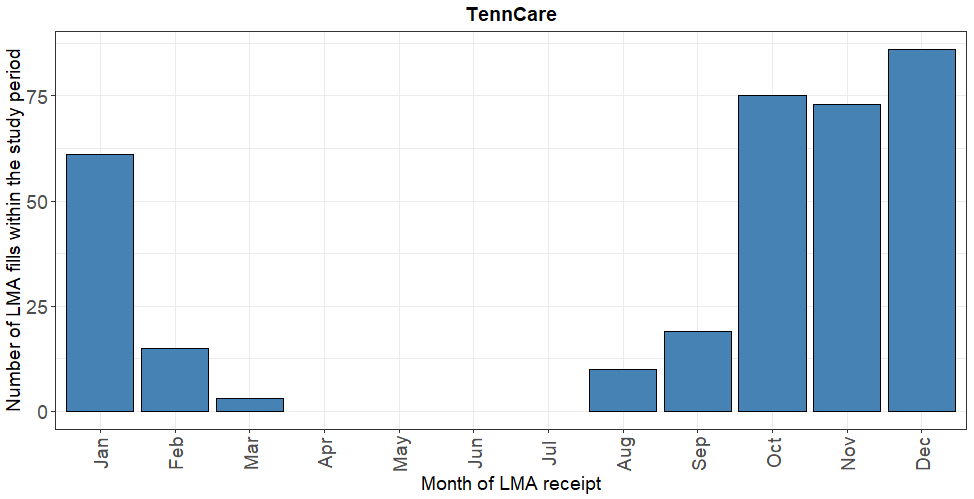

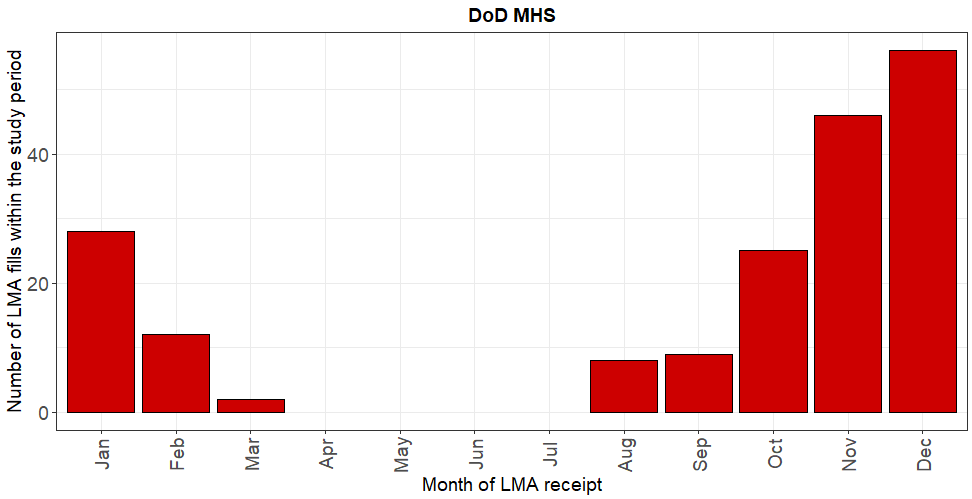

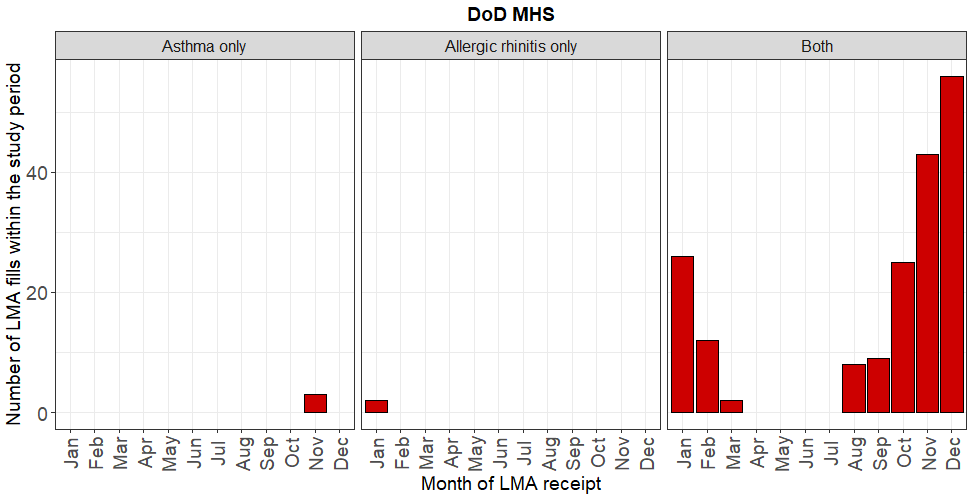
Figure S9. Leukotriene modifying agent (LMA) seasonality for Tennessee Medicaid (TennCare) and Department of Defense Military Health System (DoD MHS) populations included in the case-time-control design.

Asthma and/or allergic rhinitis status was determined at the start of the reference period.


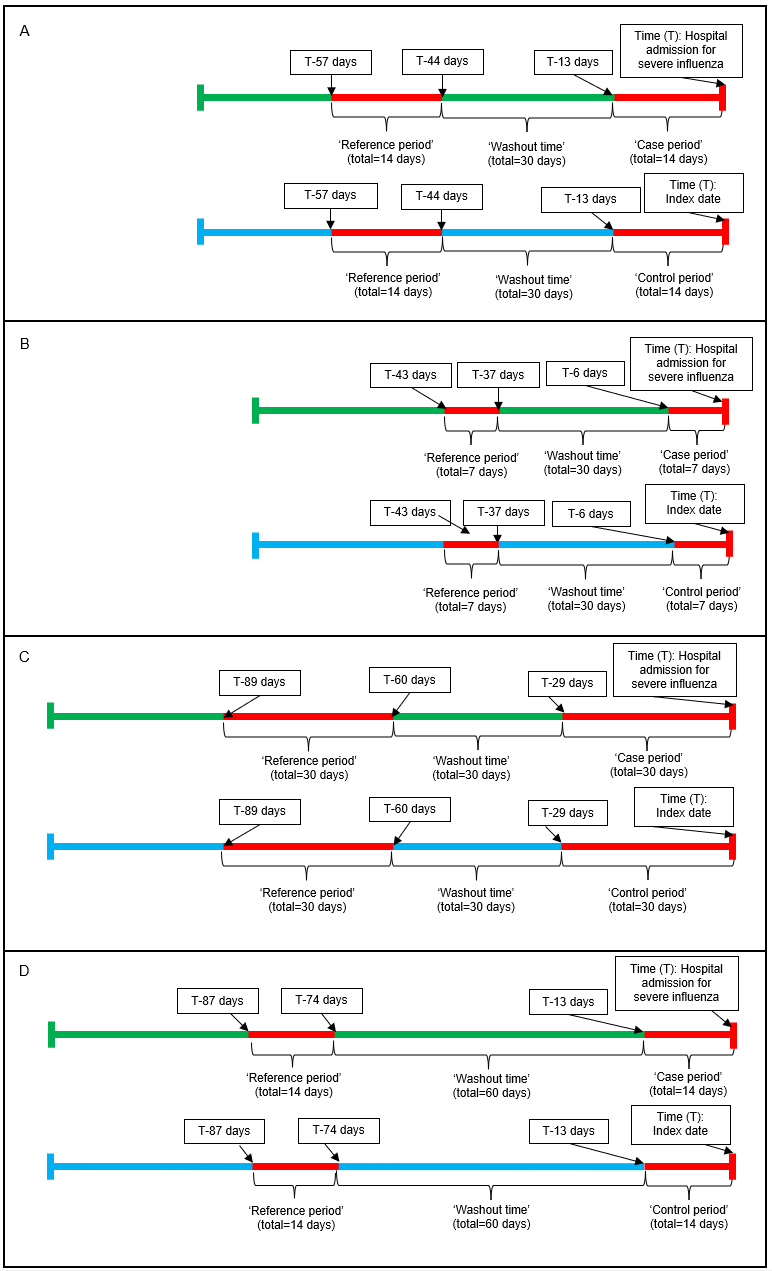
Figure S10**.** Ascertainment of case or control periods, reference periods, and washout time for the A) primary case-time-control design, B) sensitivity analysis shortening case or control and reference periods to 7 days, C) sensitivity analysis lengthening case or control and reference periods to 30 days, and D) sensitivity analysis lengthening washout time to 60 days.


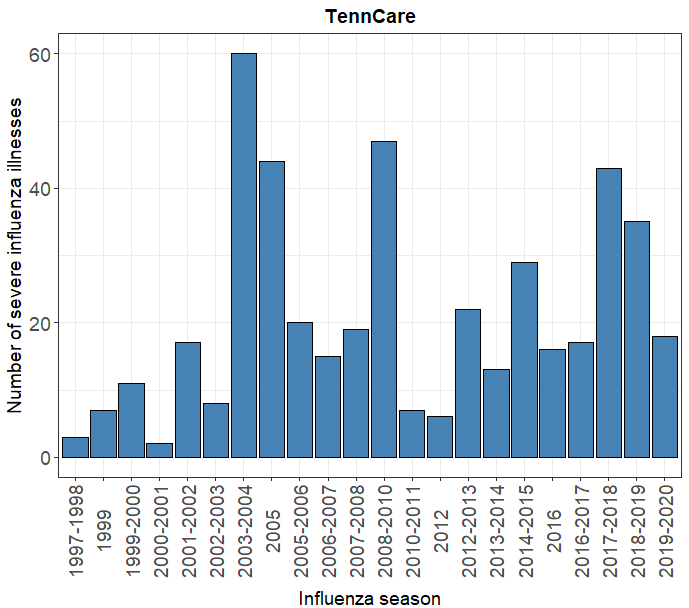
Figure S11**.** Number of severe influenza illnesses by influenza season and population for the marginal structural model analysis.

**
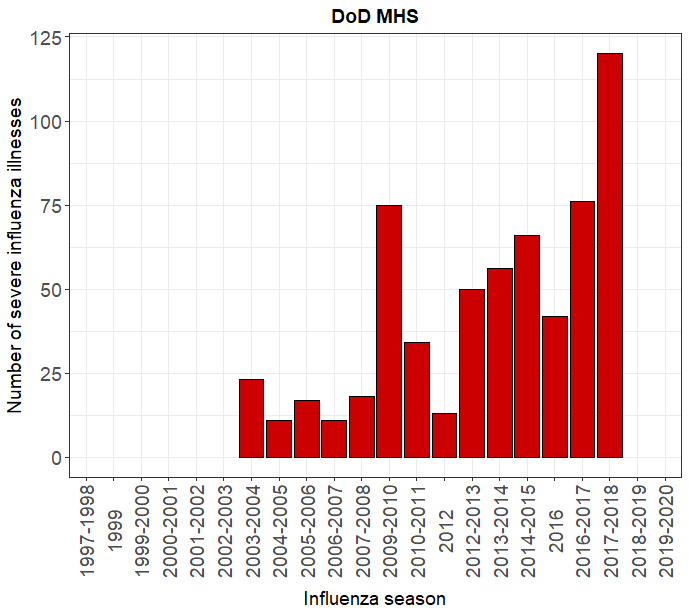
**

Earliest and latest influenza seasons were 1997-1998 and 2019-2020 for TennCare and 2003-2004 and 2017-2018 for the DoD MHS, respectively. Influenza season start and end dates were defined using influenza positive test data collected by the World Health Organization and the National Respiratory Enteric Virus Surveillance System for public health and clinical laboratories in the United States. Data were aggregated by Census Region and Morbidity and Mortality Weekly Report week to calculate the test positivity proportion for each Census Region-week. Influenza season start dates were defined as the second of two consecutive weeks where model-smoothed test positivity rate was >5% and influenza season end dates were defined as the second of two consecutive weeks where model-smoothed test positivity rate was <5%. Influenza season start and end dates from the South Census Region were used for the TennCare population, and US-wide influenza season start and end dates were used for the DoD MHS population. US-wide influenza season start and end dates were identified by the median influenza season start and end dates from all four Census Regions.

Figure S12**.** A) Association between leukotriene modifying agent use and severe influenza illness removing cases with a leukotriene modifying agent fill within seven days of hospital admission for severe influenza illness and their matched controls using the case-time-control design, B) 2x2 tables of crude odds ratio calculations.

**
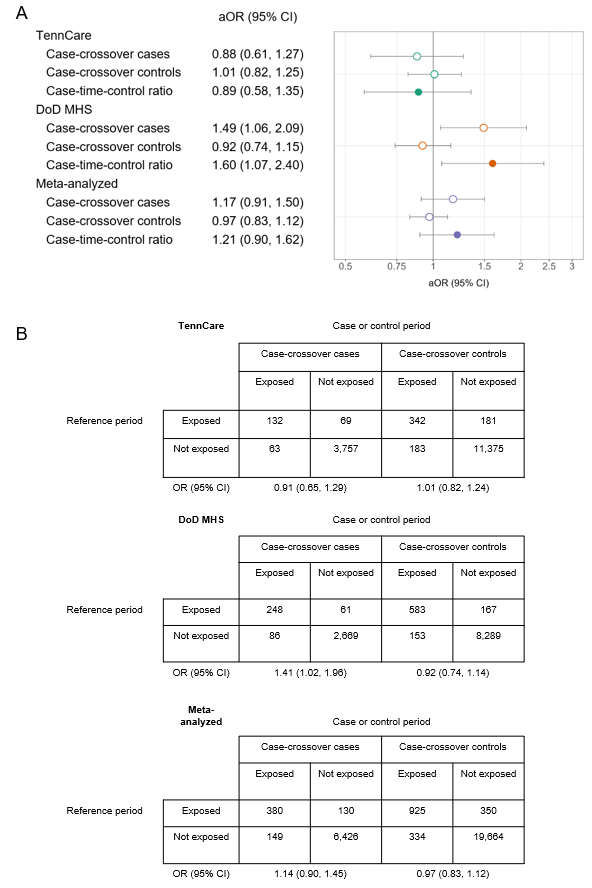
**

aOR, adjusted odds ratio; OR, odds ratio; CI, confidence interval. Matched (on subject) crude odds ratios for the case individuals and control individuals were calculated separately as the ratio of the number of case individuals and control individuals exposed only during the case or control period to the number of case individuals and control individuals exposed only during the reference period (ratio of discordant pairs) using conditional logistic regression. The case-time-control ratio was then subsequently calculated by including a case status by period interaction term in the conditional logistic regression model. Adjusted odds ratios were similarly calculated with the addition of variables capturing asthma control. Results were meta-analyzed using fixed-effects inverse variance models.

Figure S13. A) Primary and sensitivity analyses varying period widths among TennCare enrollees included in the case-time-control design and 2x2 tables of crude odds ratio calculations, B) Primary and sensitivity analyses varying period widths among DoD MHS enrollees included in the case-time-control design and 2x2 tables of crude odds ratio calculations, C) Primary and sensitivity analyses varying period widths among TennCare and DoD MHS enrollees (meta-analyzed) included in the case-time-control design and 2x2 tables of crude odds ratio calculations.


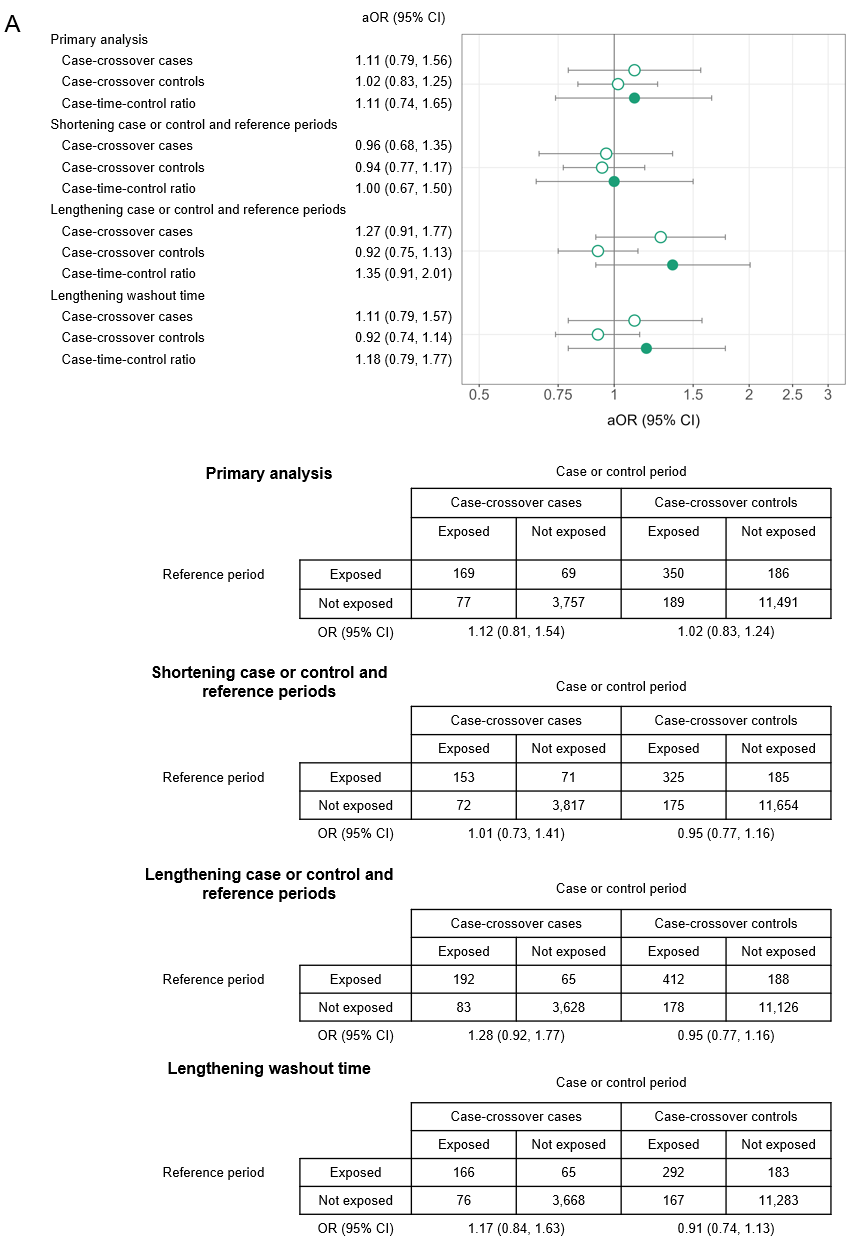


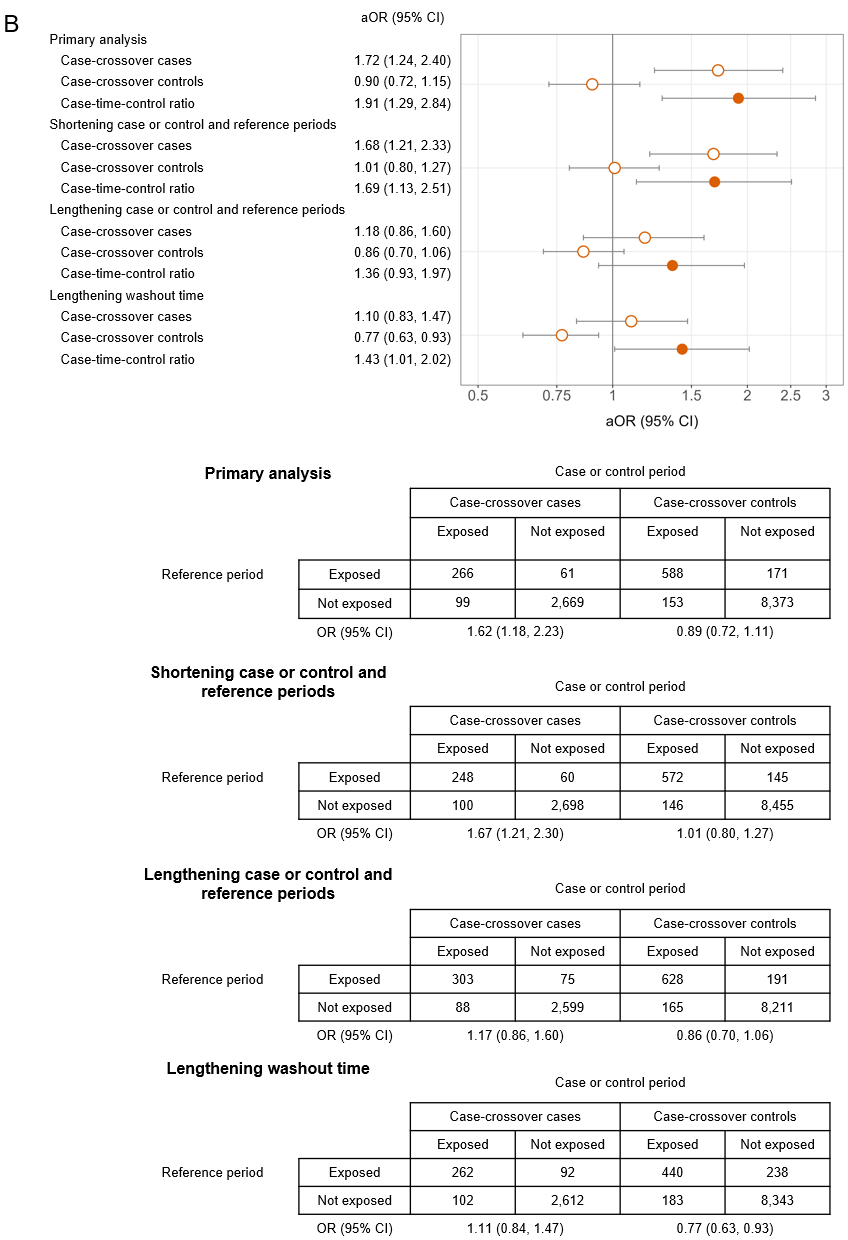


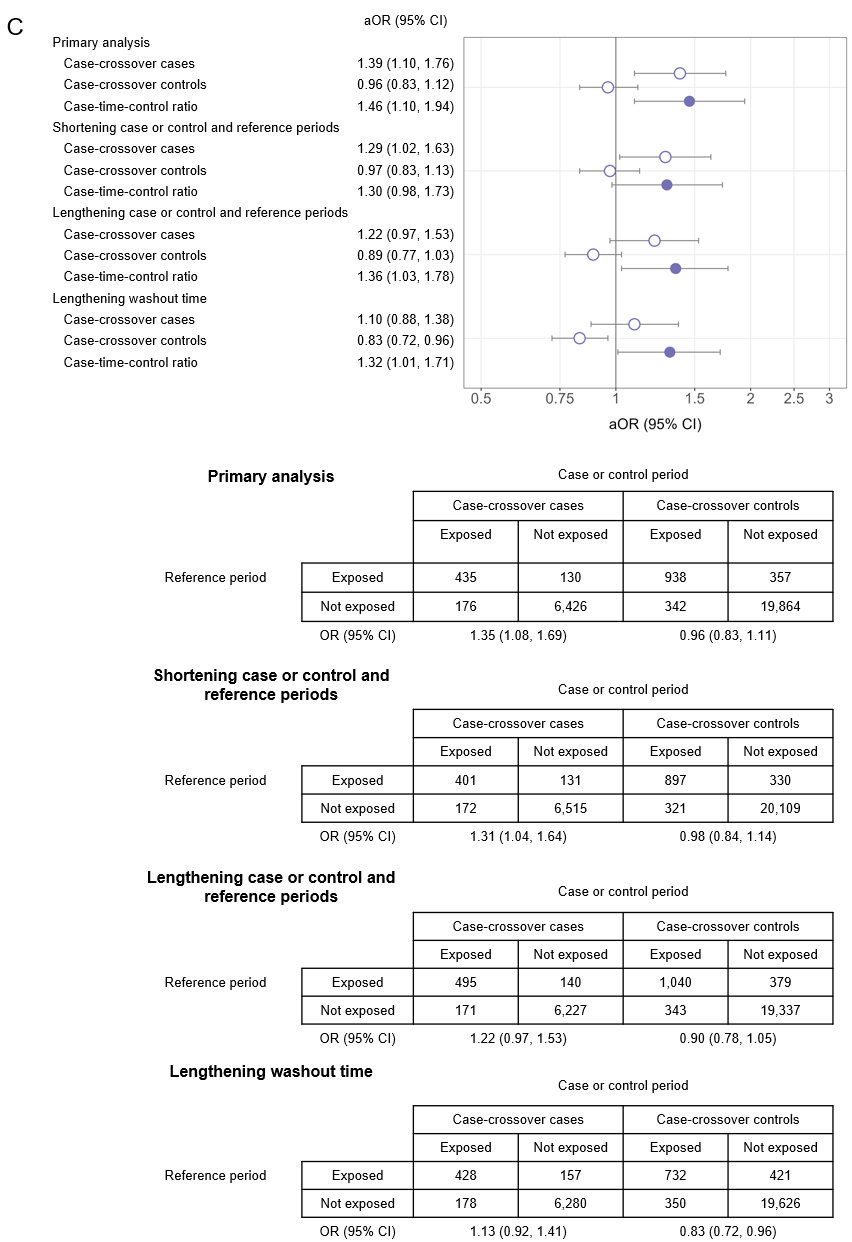


aOR, adjusted odds ratio; OR, odds ratio; CI, confidence interval. Matched (on subject) crude odds ratios for the case individuals and control individuals were calculated separately as the ratio of the number of case individuals and control individuals exposed only during the case or control period to the number of case individuals and control individuals exposed only during the reference period (ratio of discordant pairs) using conditional logistic regression. The case-time-control ratio was then subsequently calculated by including a case status by period interaction term in the conditional logistic regression model. Adjusted odds ratios were similarly calculated with the addition of variables capturing asthma control. Results were meta-analyzed using fixed-effects inverse variance models.

Table S19**.** Baseline, non-time-varying characteristics of individuals included in the case-time-control design.

| **Characteristic** | **TennCare** | **DoD MHS** |
| --- | --- | --- |
| Number of individuals, N | 16,204 | 12,368 |
| Number of periods, N | 32,576 | 24,760 |
| Birth year^a,b^, N (% individuals) |  |  |
| 1911-1923 | 331 (2) | 17 (0) |
| 1924-1936 | 885 (5) | 397 (3) |
| 1937-1949 | 1,311 (8) | 941 (8) |
| 1950-1962 | 2,183 (13) | 2,419 (20) |
| 1963-1975 | 2,151 (13) | 2,086 (17) |
| 1976-1988 | 2,685 (17) | 1,765 (14) |
| 1989-2001 | 4,107 (25) | 2,602 (21) |
| 2002-2017 | 2,551 (16) | 2,141 (17) |
| Race and ethnicity^a,c^, N (% individuals) |  |  |
| American Indian | 23 (0) | -- |
| Asian | 30 (0) | -- |
| Black | 2,994 (18) | -- |
| Hispanic/Latin | 74 (0) | -- |
| Other/Unknown | 4,123 (25) | -- |
| Southeast Asian | 35 (0) | -- |
| White | 8,925 (55) | -- |
| Sex^a,d^, N (% individuals) |  | -- |
| Female | 9,988 (62) | 6,641 (54) |
| Male | 6,216 (39) | 5,710 (46) |
| Influenza season^a,e^, N (% individuals) |  |  |
| 1994-1995 – 1996-1997 | 352 (2) | -- |
| 1997-1998 – 1999-2000 | 2,271 (14) | -- |
| 2000-2001 – 2002-2003 | 1,373 (8) | 48 (0) |
| 2003-2004 – 2005-2006 | 3,942 (24) | 1,144 (9) |
| 2006-2007 – 2008-2009 | 1,078 (7) | 916 (7) |
| 2009-2010 – 2011-2012 | 1,101 (7) | 1,916 (15) |
| 2012-2013 – 2014-2015 | 1,912 (12) | 3,336 (27) |
| 2015-2016 – 2019-2020 | 4,175 (26) | 5,008 (40) |

IQR, interquartile range.

^a^Defined at enrollment.

^b^Earliest and latest birth years were 1911 and 2017 for TennCare and 1919 and 2015 for the DoD MHS, respectively.

^c^Race and ethnicity were missing for 61% of individuals in the DoD MHS population, and is, thus, not shown. Race and ethnicity categories listed were those defined by TennCare. Data used for race and ethnicity come from a third-party data processor that creates mutually exclusive categories of race and ethnicity. This grouping may not reflect other sources of Medicaid data in Tennessee (like T-MSIS).

^e^Data missing for 0.1% of individuals included in the DoD MHS population.

^f^Earliest and latest influenza seasons were 1994-1995 and 2019-2020 for TennCare and 2002-2003 and 2017-2018 for the DoD MHS, respectively.

Table S20. Results from case-time-control design.

| **TennCare** | | | | |
| --- | --- | --- | --- | --- |
| **Case-crossover cases** | | | | |
| LMA exposure definition | # with LMA use during case period and no LMA use during reference period | # with no LMA use during case period and LMA use during reference period | OR (95% CI) | aOR (95% CI) |
| PDC=0, 0; else, 1 | 77 | 69 | 1.12 (0.81, 1.54) | 1.11 (0.79, 1.56) |
| PDC<0.2, 0; else, 1 | 69 | 67 | 1.03 (0.74, 1.44) | 1.01 (0.71, 1.45) |
| PDC<0.5, 0; else, 1 | 60 | 64 | 0.94 (0.66, 1.33) | 0.89 (0.61, 1.29) |
| **Case-crossover controls** | | | | |
| LMA exposure definition | # with LMA use during control period and no LMA use during reference period | # with no LMA use during control period and LMA use during reference period | OR (95% CI) | aOR (95% CI) |
| PDC=0, 0; else, 1 | 189 | 186 | 1.02 (0.83, 1.24) | 1.02 (0.83, 1.25) |
| PDC<0.2, 0; else, 1 | 178 | 188 | 0.95 (0.77, 1.16) | 0.94 (0.76, 1.16) |
| PDC<0.5, 0; else, 1 | 167 | 172 | 0.97 (0.78, 1.20) | 0.98 (0.79, 1.22) |
| **Case-time-control ratio** | | | | |
| LMA exposure definition | OR (95% CI) | | aOR (95% CI) | |
| PDC=0, 0; else, 1 | 1.10 (0.75, 1.61) | | 1.11 (0.74, 1.65) | |
| PDC<0.2, 0; else, 1 | 1.09 (0.73, 1.61) | | 1.10 (0.73, 1.66) | |
| PDC<0.5, 0; else, 1 | 0.97 (0.64, 1.46) | | 0.94 (0.61, 1.45) | |
| **DoD MHS** | | | | |
| **Case-crossover cases** | | | | |
| LMA exposure definition | # with LMA use during case period and no LMA use during reference period | # with no LMA use during case period and LMA use during reference period | OR (95% CI) | aOR (95% CI) |
| PDC=0, 0; else, 1 | 99 | 61 | 1.62 (1.18, 2.23) | 1.72 (1.24, 2.40) |
| PDC<0.2, 0; else, 1 | 99 | 63 | 1.57 (1.15, 2.16) | 1.67 (1.21, 2.32) |
| PDC<0.5, 0; else, 1 | 102 | 69 | 1.48 (1.09, 2.01) | 1.52 (1.11, 2.08) |
| **Case-crossover controls** | | | | |
| LMA exposure definition | # with LMA use during control period and no LMA use during reference period | # with no LMA use during control period and LMA use during reference period | OR (95% CI) | aOR (95% CI) |
| PDC=0, 0; else, 1 | 153 | 171 | 0.89 (0.72, 1.11) | 0.90 (0.72, 1.12) |
| PDC<0.2, 0; else, 1 | 158 | 171 | 0.92 (0.74, 1.15) | 0.93 (0.75, 1.15) |
| PDC<0.5, 0; else, 1 | 170 | 189 | 0.90 (0.73, 1.11) | 0.91 (0.74, 1.12) |
| **Case-time-control ratio** | | | | |
| LMA exposure definition | OR (95% CI) | | aOR (95% CI) | |
| PDC=0, 0; else, 1 | 1.82 (1.23, 2.67) | | 1.91 (1.29, 2.84) | |
| PDC<0.2, 0; else, 1 | 1.70 (1.16, 2.49) | | 1.80 (1.22, 2.66) | |
| PDC<0.5, 0; else, 1 | 1.64 (1.14, 2.38) | | 1.69 (1.16, 2.45) | |
| **Meta-analyzed** | | | | |
| **Case-crossover cases** | | | | |
| LMA exposure definition | # with LMA use during case period and no LMA use during reference period | # with no LMA use during case period and LMA use during reference period | OR (95% CI) | aOR (95% CI) |
| PDC=0, 0; else, 1 | 176 | 130 | 1.35 (1.08, 1.69) | 1.39 (1.10, 1.76) |
| PDC<0.2, 0; else, 1 | 168 | 130 | 1.29 (1.02, 1.62) | 1.33 (1.05, 1.69) |
| PDC<0.5, 0; else, 1 | 162 | 133 | 1.22 (0.97, 1.53) | 1.22 (0.96, 1.55) |
| **Case-crossover controls** | | | | |
| LMA exposure definition | # with LMA use during control period and no LMA use during reference period | # with no LMA use during control period and LMA use during reference period | OR (95% CI) | aOR (95% CI) |
| PDC=0, 0; else, 1 | 342 | 357 | 0.96 (0.83, 1.11) | 0.96 (0.83, 1.12) |
| PDC<0.2, 0; else, 1 | 336 | 359 | 0.94 (0.81, 1.09) | 0.94 (0.80, 1.09) |
| PDC<0.5, 0; else, 1 | 337 | 361 | 0.93 (0.80, 1.08) | 0.94 (0.81, 1.10) |
| **Case-time-control ratio** | | | | |
| LMA exposure definition | OR (95% CI) | | aOR (95% CI) | |
| PDC=0, 0; else, 1 | 1.41 (1.07, 1.85) | | 1.46 (1.10, 1.94) | |
| PDC<0.2, 0; else, 1 | 1.37 (1.04, 1.81) | | 1.43 (1.07, 1.89) | |
| PDC<0.5, 0; else, 1 | 1.30 (0.99, 1.71) | | 1.32 (0.99, 1.75) | |

LMA, leukotriene modifying agent; OR, odds ratio; CI, confidence interval; aOR, adjusted odds ratio; PDC, proportion of days covered.

^a^The same individual could serve as a case and control.

Matched (on subject) crude odds ratios for the cases and controls were calculated separately as the ratio of the cases and controls exposed only during the case or control period to the cases and controls exposed only during the reference period (ratio of discordant pairs) using conditional logistic regression. Adjusted odds ratios were similarly calculated with the addition of variables capturing asthma control.
